# Supplementary material for: Evidence of a cascading positive tipping point towards electric vehicles
Source: Nat Commun. 2025 Dec 8;17:240. doi: 10.1038/s41467-025-66945-9 (PMC12783758; doi:10.1038/s41467-025-66945-9)
Supplement: Supplementary file 1 — Supplementary Information [file 41467_2025_66945_MOESM1_ESM.pdf]

Supplementary Information for

Evidence of a cascading positive tipping point towards electric vehicles

Jean-Francois Mercure [a,b,c,Ψ *], Aileen Lam [d,b,Ψ], Joshua E. Buxton [c], Chris A. Boulton [c], Amir Akther [c], Timothy M. Lenton [c]

[a] The University of Exeter Business School, Exeter, UK
[b] The World Bank, Washington DC, USA
[c] Global Systems Institute, University of Exeter, Exeter, UK
[d] Department of Economics, Faculty of Social Sciences, University of Macao, E21 Taipa, Macau, China
[Ψ] These authors have contributed equally
* Corresponding author j.mercure@exeter.ac.uk

Table of contents

SUPPLEMENTARY NOTE 1. EXPERIENCE CURVES 3

SUPPL. FIGURE 1 | EXPERIENCE CURVE FOR EVS. 4
SUPPL. FIGURE 2 | COST AND PRICE REDUCTIONS OF EVS IN SELECTED MODELS. 5
SUPPL. FIGURE 3 | COST-PRICE RELATIONSHIP FOR EVS. 6

SUPPLEMENTARY NOTE 2. THE FTT:POWER MODEL IN DETAIL 7

SUPPL. FIGURE 4 | HISTORICAL AND PROJECTED CAR FLEET NUMBERS BETWEEN 1995 AND 2040. 12
SUPPL. FIGURE 5 | HISTORICAL EV SALES IN 31 COUNTRIES BETWEEN 2010 AND 2022. 13
SUPPL. FIGURE 6 | EVOLUTION OF MARKET VARIETY IN LEAD MARKETS PLUS INDIA. ICEV DENOTES INTERNAL COMBUSTION ENGINE VEHICLE, WHILE EV DENOTES ELECTRIC VEHICLE, AND PHEV DENOTES PLUG-IN HYBRID ELECTRIC VEHICLE. 14
SUPPL. FIGURE 7 | PROJECTED PRICE AND OWNERSHIP COST FOR EVS AGAINST ICEVS. 15
SUPPL. FIGURE 8 | BASELINE FLEET SHARES FOR DIFFERENT TECHNOLOGIES USING FTT:TRANSPORT. 16
SUPPL. FIGURE 9 | GROWTH OF THE ELECTRIC VEHICLE MARKET SHARES BY COUNTRY BETWEEN 2010 AND 2022. 17
SUPPL. FIGURE 10 | TRAJECTORIES FOR ACHIEVING COST PARITY FOR MID-RANGE EVS IN INDIA. 18
SUPPL. FIGURE 11 | HISTORY OF EV CHARGING POINTS. 19
SUPPL. FIGURE 12 | CHARGING POINT NUMBERS PER EV AS EVS PENETRATE MARKETS. 20
SUPPL. FIGURE 13 | PROPERTIES OF VEHICLES IN THE DATABASE. 21
SUPPL. TABLE 1 | VEHICLE DATABASE COVERAGE AND SOURCES. 22
SUPPL. TABLE 2 | THE SATURATION LEVELS, DATA SOURCES, AND VALUES FOR A AND B. 22
SUPPL. TABLE 3 | DATA SOURCES 23
SUPPL. TABLE 4 | REGRESSION RESULTS FOR PERSONAL ROAD TRANSPORT DEMAND 24
SUPPL. TABLE 5 | PROPERTIES OF VEHICLES BY ENGINE AND BATTERY SIZE 24

SUPPLEMENTARY NOTE 3. DEFINING A TIPPING POINT IN SOCIO-ECONOMIC SYSTEMS 25

SUPPLEMENTARY NOTE 4. ASSUMPTIONS CONCERNING CONTEXTUAL FACTORS 26

SUPPLEMENTARY NOTE 5. THE ROLE OF MODEL VARIETY 27

SUPPL. FIGURE 14 | RELATIONSHIP BETWEEN MODEL VARIETY AND THE BREAKEVEN SUBSIDY. 27

SUPPLEMENTARY NOTE 6. ESTIMATING ABATEMENT COSTS AND THE IMPACTS OF TARIFFS 28

SUPPL. FIGURE 15 | INTERACTIONS BETWEEN POLICY INSTRUMENTS IN FTT:TRANSPORT. 29
SUPPL. FIGURE 16 | ABATEMENT COSTS ASSOCIATED WITH SCENARIO (D) OF FIGURE 5. 30
SUPPL. FIGURE 17 | IMPACTS OF TARIFFS. 30

SUPPLEMENTARY NOTE 7. SENSITIVITY ANALYSES 31

SUPPL. FIGURE 18 | COMPARISON OF PROJECTED EV STOCKS BETWEEN OUR MODEL FTT AND THE IEA. 33
SUPPL TABLE 6 | STRINGENCY OF POLICY INSTRUMENTS IN FTT MODELLING (FIG. 5 SCENARIO D) 34
SUPPL. TABLE 7 | JUSTIFICATION FOR THE VARIATIONS FOR THE SENSITIVITY ANALYSES. 35
SUPPL. TABLE 8 | SENSITIVITY ANALYSES FOR THE CURRENT TRAJECTORY SCENARIO FOR EUROPE. 36
SUPPL. TABLE 9 | SENSITIVITY ANALYSES IN THE CURRENT TRAJECTORY SCENARIO FOR THE US. 37
SUPPL. TABLE 10 | SENSITIVITY ANALYSES IN THE CURRENT TRAJECTORY SCENARIO FOR CHINA. 38
SUPPL. TABLE 11 | SENSITIVITY ANALYSES IN THE CURRENT TRAJECTORY SCENARIO FOR INDIA 39
SUPPL. TABLE 12 | SENSITIVITY ANALYSES IN THE POLICY COMBINATIONS SCENARIO FOR EUROPE 40
SUPPL. TABLE 13 | SENSITIVITY ANALYSES IN THE POLICY COMBINATIONS SCENARIO FOR THE US. 41
SUPPL. TABLE 14 | SENSITIVITY ANALYSES IN THE POLICY COMBINATIONS SCENARIO FOR CHINA 42
SUPPL. TABLE 15 | SENSITIVITY ANALYSES IN THE POLICY COMBINATIONS SCENARIO FOR INDIA 43
SUPPL TABLE 16 | SENSITIVITY ANALYSES ON THE STRINGENCY OF POLICY INSTRUMENTS, CHINA 44
SUPPL TABLE 17 | SENSITIVITY ANALYSES ON THE STRINGENCY OF POLICY INSTRUMENTS, US 45
SUPPL TABLE 18 | SENSITIVITY ANALYSES ON THE STRINGENCY OF POLICY INSTRUMENTS, EU 46
SUPPL TABLE 19 | SENSITIVITY ANALYSES ON THE STRINGENCY OF POLICY INSTRUMENTS, INDIA 47

SUPPLEMENTARY NOTE 8. EXPONENTIAL RISE IN EV SALES SHARE 48
SUPPLEMENTARY REFERENCES..48

Supplementary Note 1. Experience curves

Experience curves are sometimes defined as a percentage of decrease in cost or market price (the experience rate ER_i) for each doubling of cumulative production.[1] This can be expressed as a power-law that connects the cost or price of a technology to its cumulative production:

$$C_i(t) = C_i(t_0)\left(\frac{W_i(t)}{W_i(t_0)}\right)^{-b_i}, \qquad ER = 1 - 2^{-b_i} \tag{1}$$

Where $C_i(t)$ and $C_i(0)$ are the cost at time t and at a reference time t_0, respectively, while $W_i(t)$ and $W_i(t_0)$ is the cumulative experience at those moments. b_i is the experience curve exponent related to the experience rate (or sometimes referred to as learning rate) ER_i.

Experience curves are technically different than learning curves, although they are sometimes used interchangeably. Learning curves relate specifically to cost reductions associated with accumulated experience by the labour force producing the technology, whereas experience curves express all cost reductions related to any component of the production system.[2]

We stress that the power-law form of the experience curves never crosses zero except at infinity. In practice, experience curves do not come close to unrealistically low values either, due to the exponentially rising amount of effort required to maintain a constant rate of cost reduction. At some point in the diffusion lifecycle, it typically becomes impossible to double capacity any further, and cost reductions stall (this is the case for all mature technologies). There can never be any guarantees that the cost of any new technology achieves parity with older technologies.

Here we apply the experience curve to both electric vehicle *battery costs* and electric *vehicle prices*. Cost reductions in batteries form the largest source of vehicle manufacturing cost reduction. We reproduce with our learning rates found in the literature (Suppl. Figure 1). We measure 15-21%, variations that depend on how the linear fit is done and whether 2010 data is included or not. Our data sees an acceleration after 2011. In FTT, we use the value of 20% reported in the literature. We test the impact of varying this value by ±10% through sensitivity analyses and only find moderate effects (Suppl. Note 5).

In our model, learning curve cost reductions are assumed to become reflected into car prices. We acknowledge that production costs may not in principle necessarily become reflected into market prices as production can be cross-subsidised (using for example income from the sale of other products), or, cost reductions can be kept as profits rather than passed on into prices. Also, battery sizes tend to increases over time as battery production costs decrease.

To test this, we use EV prices and matching battery capacity between 2016 and 2023 for a subset of 14 models from our dataset in the UK, US, Germany and France. For other vehicle models, battery capacity values do not cover the entire historical period. We use battery costs per unit capacity from the literature (Figure 2 of the main paper). We establish a relationship between the two, given in Suppl. Figures 2-3.

In Suppl. Figure 2, we show that (A) as unit battery costs decline over time, (B) vehicle prices decline as well. Battery capacities increase over time (C), but (D) total battery costs (A times C) generally decline nonetheless. In (E), vehicle prices normalized by their battery capacity decline, while in (F) vehicle prices normalized by their battery costs are approximately stationary.

In Suppl. Figure 3, we show that vehicle prices scale with battery costs by a factor 5 in Europe (UK, Germany, France) and 2 in the US. This means that, not only are battery cost reductions passed on to consumers, but cost reductions achieved in the manufacturing of the rest of the vehicles are also passed on to consumers, at a similar rate (our data does not allow to distinguish the two rates). This supports our assumption that learning cost reductions in the manufacturing of EVs are reflected in vehicle prices. We assume that this continues to be the case in the coming years.

We stress that this assumption *does not* imply that EV prices must inevitably cross cost parity with ICEVs in our model, since cost reductions depend on sales, which are endogenous in our model. If sales of EVs were to decline significantly, cost reductions would also cease, which would reinforce sales declines (in a positive feedback) and could fail to achieve cost parity (as for instance happens with some PHEVs).

These dynamics are consistent with what occurred in the early days of the internal combustion vehicles.[3] This is also common in product innovation lifecycles.[4]

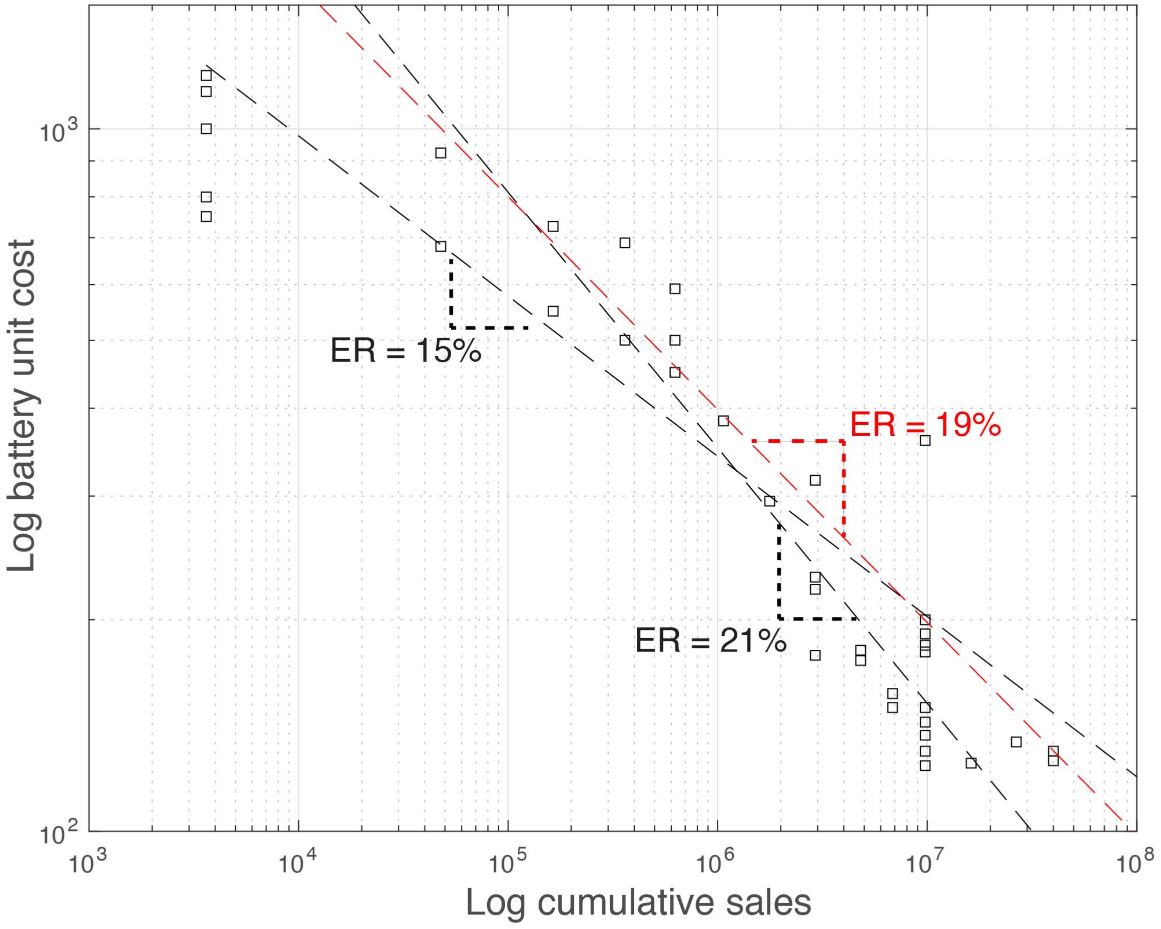

Suppl. Figure 1 | Experience curve for EVs. Estimation of the experience rate (ER) for batteries out of the data from this work (Figure 2 from the main paper). The value range between 15% and 21% agrees with the literature which report 20%. In the FTT model we adopt the literature value of 20%.

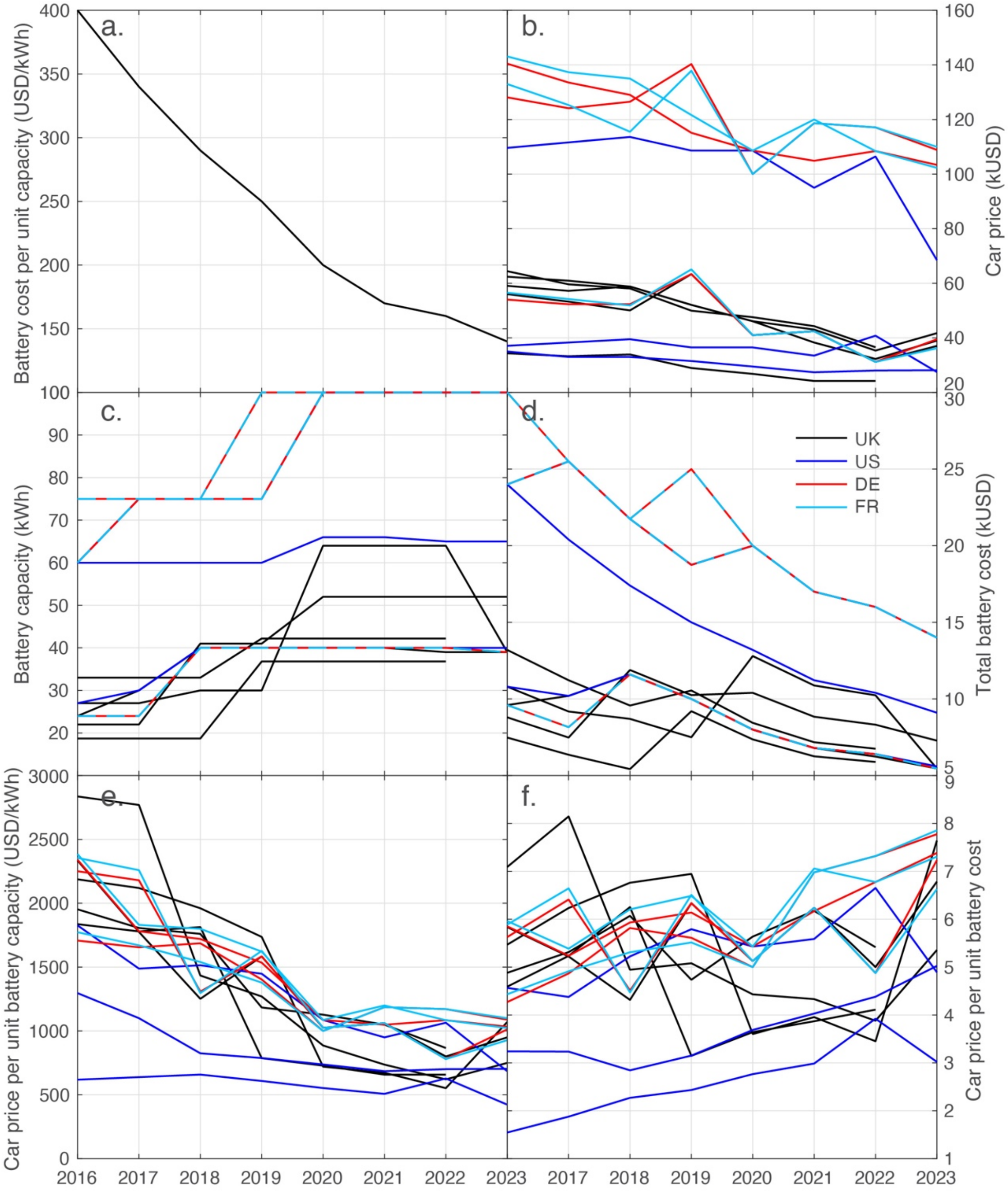

Suppl. Figure 2 | Cost and price reductions of EVs in selected models. Time series for 14 vehicle models across the UK (5), US (3), Germany (DE, 3) and France (FR, 3) used to explore the relationship between vehicle prices and battery costs. Data includes vehicle prices, battery costs per kWh and battery capacity. a) Decline in global battery costs per unit battery capacity. b) Declines in prices per region for a range of EVs c) Battery capacities of a range of EVs over time. d) Battery cost declines over time for a range of EVs. e) Declines in vehicle prices per unit of battery capacity for a range of EVs. f) Vehicle prices shown in b) normalised by battery costs shown in d) for a range of EVs, a quantity that is roughly stationary.

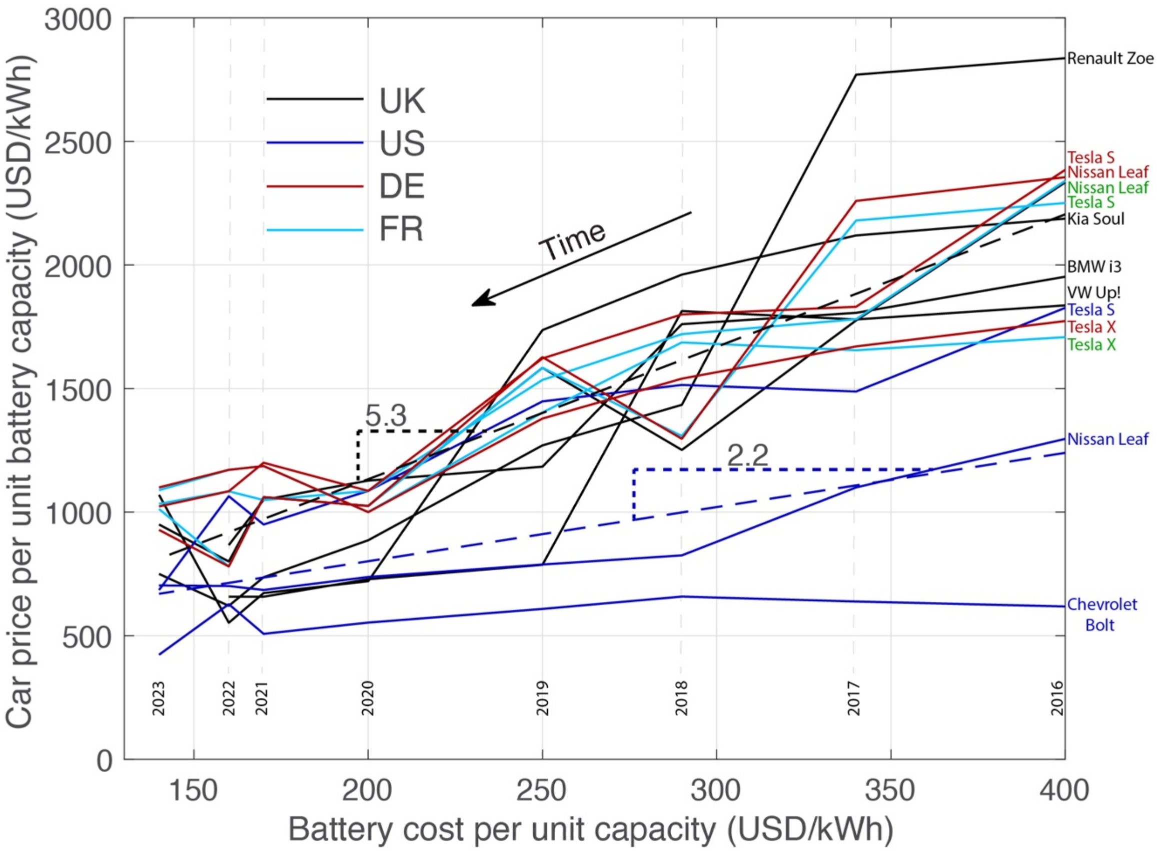

Suppl. Figure 3 | Cost-price relationship for EVs. Linear scaling between vehicle prices per unit by battery capacity (vehicle price per unit battery performance) and battery unit costs suggests that battery cost reductions are reflected into vehicle prices. Dashed lines are fits over the data for the UK, Germany (DE), France (FR) put together, and the US separately. Slopes indicate that vehicle prices in Europe are on average 5.3 times their battery costs, while in the US they are 2.2 times their battery costs.

Supplementary Note 2. The FTT:Power model in detail

This note describes the FTT:Transport model from a theoretical perspective with equations. More information can be obtained in references [5–10].

Social influence and technological diffusion

The FTT model method can be derived from an evolutionary improvement over standard discrete choice theory. This variant enables to reproduce the widely observed S-curve profile of technology diffusion, which is not obtained from the standard discrete choice model. The standard model assumes instantaneous choices with perfect information, two assumptions that the evolutionary version relaxes.

Standard discrete choice models (or multinomial logit models) study decision-making by groups of agents with non-identical preferences facing a list of options, on the basis of utility maximization when access and information is fully and equally available to all agents and decisions are instantaneous. It defines a linear random utility model, in which the utility U_i* (associated with purchasing a particular type of vehicle i in a list of n options) is expressed as a function of a number of variables *V,* such as income, gender, and distance travelled and so on, and regression parameters β and residual (stochastic noise) ε.

$$U^* = \beta_i^1 V_i^1 + \beta_i^2 V_i^2 + \beta_i^3 V_i^3 + \beta_i^4 V_i^4 + \cdots + \epsilon_i \quad (2)$$

Identifying the probability that particular option i is chosen over all other options, we get

$$P(U > \max[U_1, U_2, U_3, \dots, U_4]) = P(U > U_1) * P(U > U_2) * P(U > U_3) * \dots * P(U > U_n) \quad (3)$$

Assuming that utility frequencies for each menu options are described by Gumbel distributions (double exponentials with width parameter σ), this leads to the standard multinomial logit model (MNL[11]), which expresses the frequency of relative preferences in the group of agents:

$$P_i = \frac{e^{\frac{U_i}{\sigma}}}{\sum_j e^{\frac{U_j}{\sigma}}} \quad (4)$$

In the case of cars, the standard MNL model takes the probabilistic choice P_i as equal to market shares of each technological option as the market settles, in equilibrium, following changes in variables. It is in that sense that choices are instantaneously reflected in vehicle fleet additions. Importantly, in an MNL model, if variables do not change, P_i remains unchanged, as endogenous diffusion is not modelled (costs have to change for diffusion to happen). Furthermore, the popularity or visibility of a vehicle type has no impact on its diffusion, which means that the entire population could in principle, instantaneously buy into something that was largely unknown previously, given a sufficiently large subsidy. *This is unrealistic and not suitable to model diffusion trajectories*.

The standard MNL model does not reproduce observed S-curves of diffusion, unless it is very precisely and deliberately parameterized to do so (for example, by choosing price trajectories that generate such S-curves). The source of the problem is that the MNL does not include interactions between agents, notably, how early adopters induce adoptions from mainstream adopters.[7]

In FTT, we include social influence (agents influencing each other's purchase choices) as the basis upon which agents build information that they use to make informed choices, then the list of options seen by each agent as available to them differs. It is known empirically that this is what can produce the S-curve pattern, where the more a new technology diffuses, the more it becomes able to further diffuse, via visual influence and information contagion.[12,13] We therefore assume

that the more a product is used, the larger the number of agents will consider it within their choice list.

We assume that the relative frequency of agents having knowledge of product i is the share of the market occupied by that product. We re-evaluate Suppl. Equation (3) by weighing the factors according to their frequency of occurrence, that is $N_1, N_2 \ldots, N_n$ the numbers of agents who use each technology in the set, for a total N. We obtain:

$$P(U > \max[U_1, U_2, U_3, \ldots, U_4])^N = P(U > U_1)^{N_1} * P(U > U_2)^{N_2} * P(U > U_3)^{N_3} * \ldots * P(U > U_n)^{N_n} \quad (5)$$

Taking market share $Si{=}Ni/N$, then we have:

$$P(U > \max[U_1, U_2, U_3, \ldots, U_4]) = P(U > U_1)^{S_1} * P(U > U_2)^{S_2} * P(U > U_3)^{S_3} * \ldots * P(U > U_n)^{S_n} \quad (6)$$

The MNL variant that includes social influence is:

$$P_i = \frac{S_i e^{\frac{U_i}{\sigma}}}{\sum_j S_j e^{\frac{U_j}{\sigma}}} \quad (7)$$

Since here preferences depend recursively on preferences, P_i cannot be equal to market shares (unless both P_i and S_i equal 1 for one technology and zero for all others, which we exclude by construction). In reality, the system takes too long to reach this sort of equilibrium. This is the nature of S-curves, where social influence operates over long timescales (it typically takes years for a product to diffuse to saturation, during which the system never reaches equilibrium).

While preferences P_i are instantaneous, car purchases actually happen at a rate τ^{-1}, which expresses how frequently new cars enter the fleet (τ is the vehicle life expectancy). The rate of change of market shares is proportional to purchase preferences multiplied by the purchasing rate, minus depreciation:

$$\frac{dS_i}{dt} = \frac{1}{\tau}\left(\frac{S_i e^{\frac{U_i}{\sigma}}}{\sum_j S_j e^{\frac{U_j}{\sigma}}} - S_i\right) \quad (8)$$

This is a form of replicator dynamics equation, and evolutionary selection system in which the frequency of technology adoption is proportional to the technology's pre-existing prevalence (or visual presence) despite the standard logit preference factor.

Suppl. equation (8) can be mathematically transformed into a standard Lotka-Volterra set of coupled diffusion equations for competing species. It generates S-shaped diffusion curves consistent with the very well-established empirical technology diffusion literature.[14]

$$\frac{dS_i}{dt} = \sum_j S_i S_j \left(A_{ij} F_{ij} - A_{ji} F_{ji}\right) \quad (9)$$

Where F_{ij} is a binary logit (or logistic) function of the utility difference

$$F_{ij} = \frac{1}{1 + e^{\frac{(U_j - U_i)}{\sigma_{ij}}}}, \qquad \sigma_{ij} = \sqrt{\sigma_i^2 + \sigma_j^2} \quad (10)$$

The Lotka-Volterra, or replicator equation, has been widely used in theoretical biology to study the evolution of populations in interacting ecosystems. In FTT, utilities are proxied using the negative of costs, described below. The trajectory of S-curves is calibrated to historical data.

The decision-making module in FTT:Transport

Purchasing decisions are based in the model on perceived cost comparisons. We assume that distributions of perceived costs correspond to our measured distributions of observed costs, as per Figure 3 of the main text. We assume that agents compare vehicles on the basis of a single metric, the Levelised Cost of Transportation (LCOT), which we construct to encompass all relevant considerations in buying a vehicle (purchase price, operational costs, performance, looks etc). However, we assume that this metric is distributed across the population. Some of the components are measurable, others are not and must be inferred. All cost components are assumed always distributed, and we measure those distributions via large data gathering exercises.

$$LCOT_i = \frac{(I_i - EVS_i)}{CF_i} + \frac{\sum_t \frac{\frac{RT_i}{CF_i} + (F_i(t) + FT_i(t)) * FE_i * Dist_i + MR_i}{(1+r)^t}}{\sum_t \frac{1}{(1+r)^t}} \quad (11)$$

I_i, F_i, *and* MR_i are the average car price (in USD), fuel cost (in USD/litre, with fuel economy FE_i), and maintenance cost (in USD/km), respectively, for technology category *i* under its measured distributions for these quantities. EVS_i represents EV subsidies paid to car purchasers (and therefore, negative cost) at the purchase time. FT_i is the fuel tax, in USD/litre. The fuel cost depends on the fuel economy parameter $FE_i(t)$ and the distance travelled each year ($Dist_t$). $RT_i(t)$ is the annual registration tax, which is vehicle and class-specific, paid by car owners once per year. CF_i is the load factor, in km/y. Costs are discounted with rate *r* according to when, in the vehicle lifecycle, costs are incurred, where the purchase cost happens at *t=0* and therefore not discounted. Other unmeasurable costs (the intangibles) are discussed below.

The generalised cost and the intangibles

As inferred from the price distribution of sales, transport costs are not the only factors that consumers consider when purchasing a vehicle. Many additional aspects (e.g., comfort and luxury) are valued by consumers, of which we have little information beyond the price distribution of what is purchased. We keep in mind that technologies have different pecuniary costs, particularly across engine size classes; despite this, higher costs appear compensated by higher benefits, such that higher cost luxury vehicles maintain market shares. Notably, EVs can benefit from an environmental value that some consumers may be willing to pay for.

Were we to simulate technology diffusion based on bare LCOT distribution comparisons, the lowest LCOT technologies would diffuse more successfully, Which is not consistent with our historical data. The components missing in the LCOT to make correct forecasts—for instance, comfort, acceleration, and style— are what we call the "intangibles". We define "intangibles" for this model as the difference between the generalized cost, the cost our model needs to reproduce the observed diffusion trends, and the LCOT, as calculated from purely pecuniary vehicle properties for which we have data. The value of the intangibles, γ_i, is an empirically estimated parameter obtained from making the FTT diffusion trajectory match the trajectory observed in our historical data, at the year of the start of the simulation.

Costs are lognormally distributed (normally distributed in log space), therefore we assume that it is the log of the costs that are compared by agents. Incorporating the ingantibles γ_i in log space yields the following (see the Supplementary Information in [8]):

$$C_i = \ln\left(\frac{LCOT_i^2}{\sqrt{LCOT_i^2 + \Delta LCOT_i^2}}\right) + \gamma_i$$

$$\sigma_i = \sqrt{\ln\left(1 + \frac{\Delta LCOT_i^2}{LCOT_i^2}\right)} \qquad (12)$$

Where the $\Delta LCOT_i$ is the standard deviation of the LCOT for category *i*. This transformation relates to comparing costs in log space using parameters measured in dollar space.

A unique set of γ_i exists that minimises the difference in diffusion trajectory slope at the start of the simulation between the simulation and historical data, which is minimised. To ensure it is correctly identified, each estimation is checked visually. This is done for each technology in every region to ensure that the parameters are not spurious. We find that γ_i values follow what should be expected: luxury models have large negative values (large intangible benefits). Since generalized cost differences already exist in the baseline, diffusion trends exist in the baseline, a fact that is observed in the data (e.g. the pace of diffusion of EVs), and the determination of the γ_i parameters is of primary importance. A sensitivity analysis has been carried out to assess how the uncertainties in γ_i affect the simulations of the FTT-Transport model (Suppl. Note 5).

Car population projections

Car ownership models are used to forecast transport demand, energy consumption, and emission levels. Among the different model types, one of the most well-known approaches is an econometric estimation of an income-car stock model based on a logistic function[15,16]. Historically, GDP growth and economic development are associated with an increase in vehicle ownership. Past studies have made projections of passenger car ownership based on GDP[17–19].

The Gompertz curve is an S-shaped growth curve (independent from the S-curve for diffusion) that relates per capita vehicle ownership to GDP per capita. While vehicle scrappage is not explicitly included, this relationship has been tested empirically to represent the growth trend of vehicle stock[16]. We examine trends in the growth of vehicle stocks for a large sample of countries (including EU countries, US, China, India) and employ the Gompertz function to estimate the relationship between the number of vehicles and per capita income.

Following previous studies, we estimate the vehicle stock with a Gompertz model:

$$V_{i,t} = V_i^* e^{\alpha e^{\beta EF_{i,t}}}, \qquad (13)$$

which is equivalent to regressing the following linearly:

$$\ln\left(\ln\left(\frac{V_{i,t}}{V_i^*}\right)\right) = \ln(\alpha) + \beta EF_{i,t} \qquad (14)$$

Here, $V_{i,t}$ represents the vehicle ownership (vehicles per 1000 people) of country *i* in year *t*, V_i^* is a saturation level and *EFi,t* is the average per capita income. The parameter α determines car stock demands at zero income levels, and the parameter β determines the shape of the growth curve. We find these values by regressing $\ln\left(\ln\left(\frac{V_{i,t}}{V_{i,t}^*}\right)\right)$ against $EF_{i,t}$.

Suppl. Table 1 provides data sources. The results are shown in Suppl. Table 2. Suppl. Figure 4 shows the historical fleet sizes and the projected vehicle stocks for Europe, the US, China and India under the Shared Socio-Economic Pathways SSP2. These projections have been used to

calibrate total vehicle numbers in FTT:Transport, whereas the replicator equation (Suppl. Equation (9)) determines the technological composition of this total.

Projection of the demand for transport services

The demand for Personal Light Duty Vehicle (PLDV) services (used in FTT:Transport) is driven by income, population, urban density, family structure and other demographic factors. Studies have also found induced and rebound effects on the demand for passenger car transport[20,21]. More specifically, they find that the demand for transport increases with economic and infrastructure development. Hence, it is important to consider the elasticity of demand for transport in relation to fuel prices, energy efficiency, and road accessibility in the estimation and projection of the demand for PLDV services.

The demand estimation consists of two parts. The first is the construction of an econometric model that predicts the demand for PLDVs (in km per vehicle) using fuel prices, income, urbanisation, road infrastructure, urban density, and fuel economy. Then we use the econometric model to predict the future private passenger vehicle transport demand (per vehicle). In the second part, we develop a model for vehicle stock and project future car ownership (previous section), which is then used to make projections for the total demand for PLDVs.

The empirical model specifies kilometres driven per PLDV in the country *i* as a function of GDP per capita[22] (*Y*), fuel cost in terms of the oil price[23,24] (*FP*), and a group of variables, including urbanization[22,25] (*U*), road lengths[23,25] (*M*), urban density[26] (*UD*), and fuel economy[27] (*FE*). The data sources are presented in Suppl. Table 3. We estimate a dynamic model because efficiency improvements and fuel price changes take time, and static models may not capture adequately the long-run adjustments of transport demand. The dynamic model we specify captures the historical trend of passenger vehicle travel demand.

We use pooled Ordinary Least Squares (OLS), Fixed Effects (FE), and the Arellano-Bond GMM model. Estimates are presented in Suppl. Table 4. Consistent with existing studies, the results show that oil prices, urbanisation, road mileage, population density, and fuel efficiency have a significant effect on road transport demand. Income does not significantly affect the distances travelled by car per year probably because, as income increases, users purchase more vehicles instead of travelling more in each car. The coefficient results show that road accessibility has a positive effect on road transport demand, while travel demand decreases by 1.5% when the oil price increases by 10%. As countries become more urbanised, people take advantage of the public infrastructure when they are in cities. Hence, we find that distance per car falls as countries become more urbanised and that distance per car increases as more roads are built (induced demand). Fuel efficiency improvements will result in a transport increase, although the effect is small (travel demand increases by 0.3% when fuel efficiency improves by 10%).

Vehicle market segmentation

Consistent with the definition of Eurostat, ICEVs (including HEVs and PHEVs) are segmented into three engine size categories: ‘Econ’ denotes cars with engine sizes smaller or equal to 1400cc; ‘Mid’ denotes cars with engine sizes larger than 1400cc and smaller than 2000cc, and ‘Lux’ denotes cars with engine sizes larger than 2000cc. For the present study, an original database detailing the technological profile of cars and populations was built, with data sources presented in Suppl. Table 1. Suppl. Figure 13 shows the scatter plots with univariate correlations between engine sizes and powers for all new vehicles sold in Europe, the US and China in 2020. In the bubble graphs, one circle is shown per model, of which the size is scaled with the sales number for each model.

Not having an internal combustion engine, EVs are segmented according to their ranges or battery sizes. Since larger battery capacities lead to longer ranges and larger power output (Suppl. Figure

13), we use the battery capacity which affects the key performance of EVs as the parameter used to create EV market segments. There is a degree of uncertainty in the vehicle power output of the same engine and battery size bracket (see Suppl. Table 5). We define equivalent ICEVs and EVs as those that have a similar power output range. Accordingly, 'Econ' denotes EVs with battery capacities smaller or equal to 30kWh; 'Mid' denotes EVs with battery capacities larger than 30kWh and smaller than 70KWh; 'Lux' denotes EVs with battery capacities larger than 70kWh.

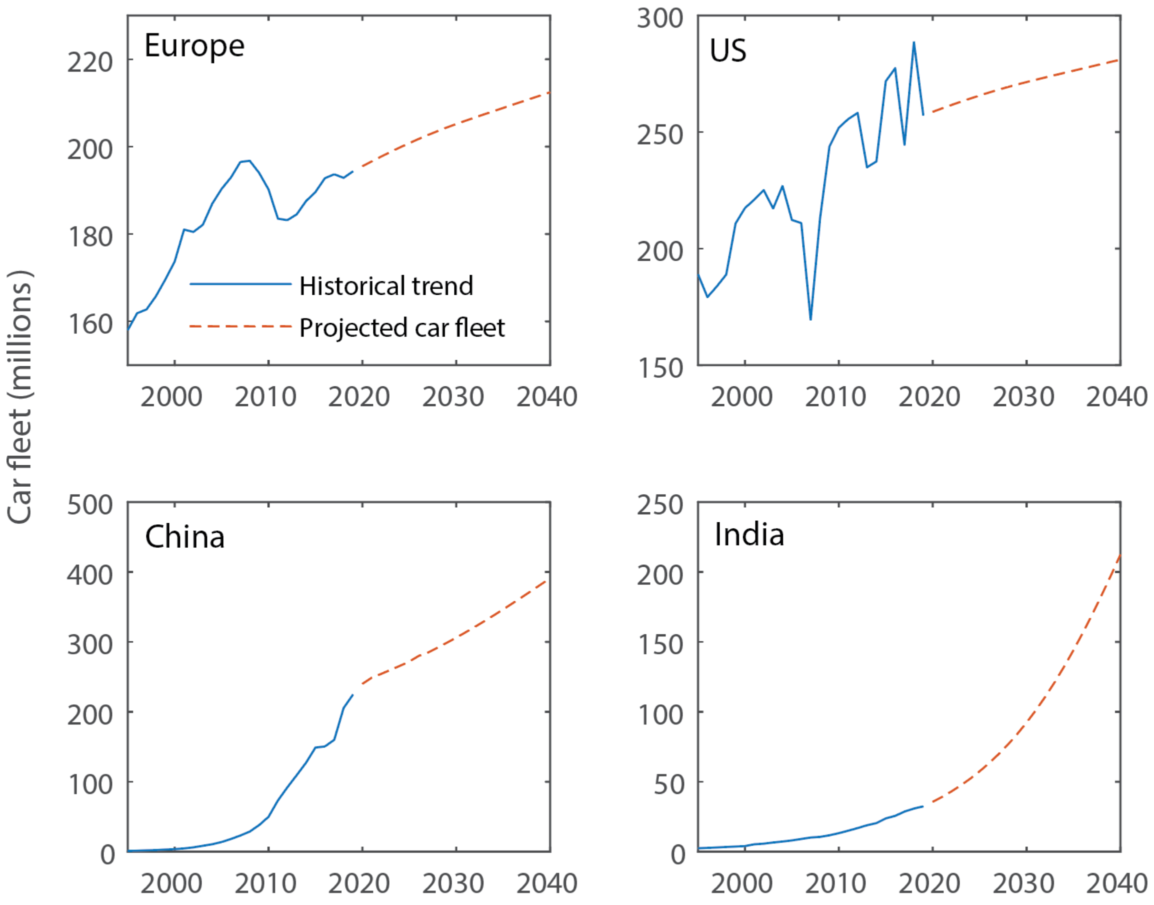

Suppl. Figure 4 | Historical and projected car fleet numbers between 1995 and 2040. Historical data are in solid lines, while projections are in dotted lines. The projections are done using equation Suppl. Equation (13). Vehicle numbers tend to saturate with economic development, urbanisatoin and the density of vehicles on roads.

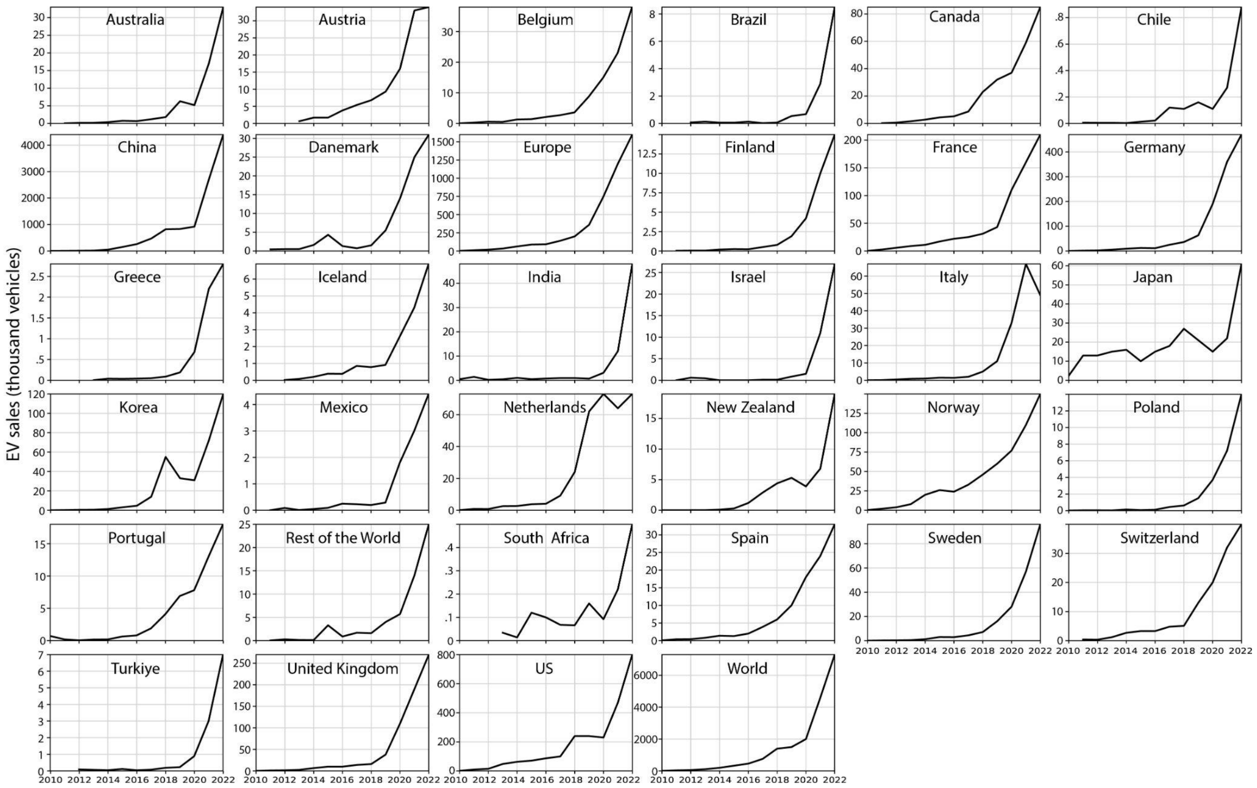

Suppl. Figure 5 | Historical EV sales in 31 countries between 2010 and 2022. The trends rise exponentially, notably with R2 greater than 0.8 for the US, EU countries and China. The data originates from the International Energy Agency.

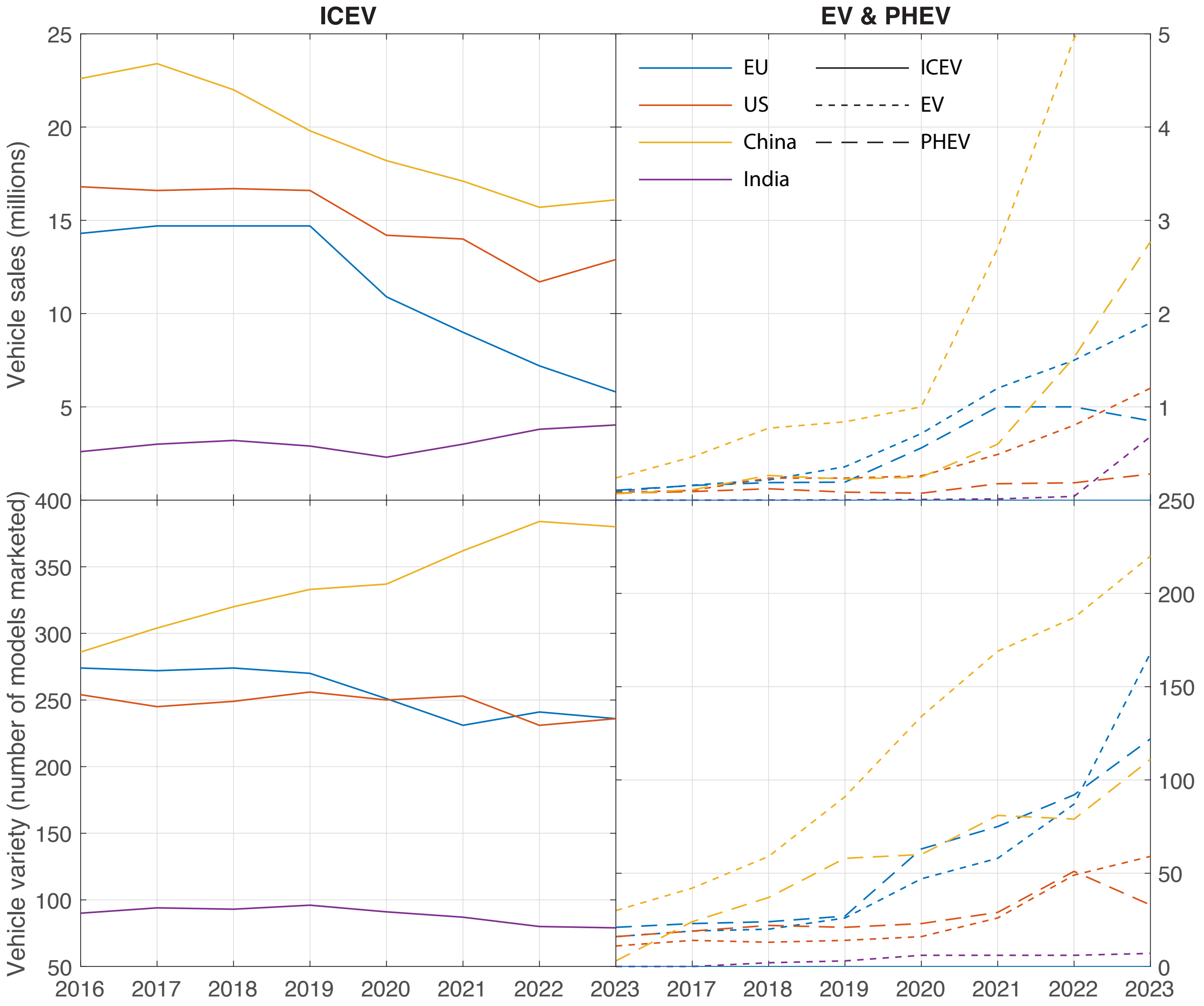

Suppl. Figure 6 | Evolution of market variety in lead markets plus India. ICEV sales have started to decline in around 2019, while EVs and PHEVs have exploded exponentially. The variety of ICEVs has stagnated or begun to decline, while the variety of EVs and PHEVs has exploded exponentially too. ICEV denotes Internal Combustion Engine Vehicle, while EV denotes electric vehicle, and PHEV denotes Plug-in Hybrid Electric Vehicle.

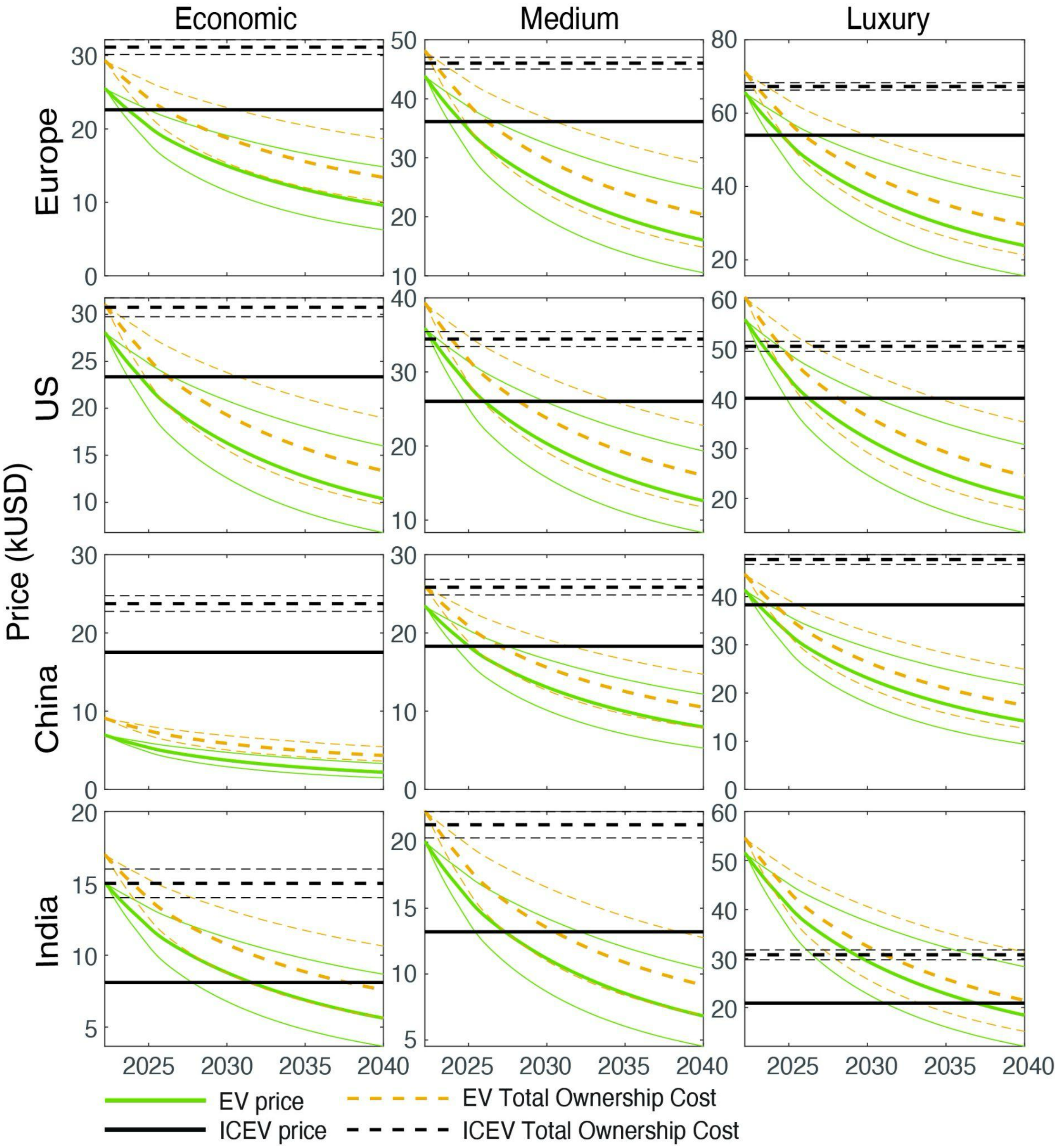

Suppl. Figure 7 | Projected price and ownership cost for EVs against ICEVs. Trajectory of total ownership costs (dashed lines) and prices (solid lines) of EVs/ICEVs, using median (thick lines) and 95% confidence range experience curve rates (thin lines). ICEV denotes Internal Combustion Engine Vehicle, while EV denotes electric vehicle.

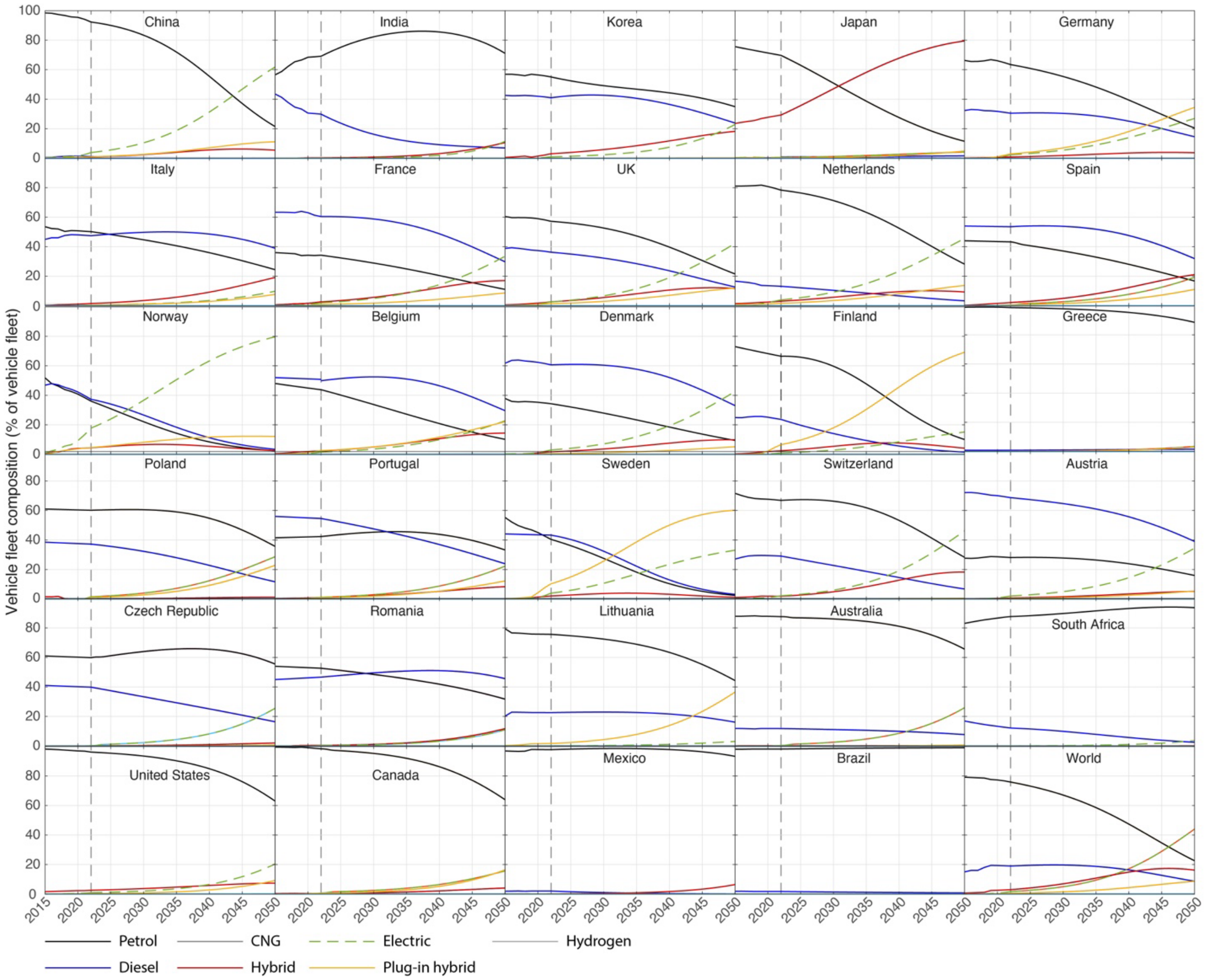

Suppl. Figure 8 | Baseline fleet shares for different technologies using FTT:Transport. These projections are made on the basis of requiring the model to reproduce rates of growth observed in the most recent historical data in the early years of the simulation. Trajectories are determined through the dynamic co-evolution of the diffusion of technologies and the evolution of their costs.

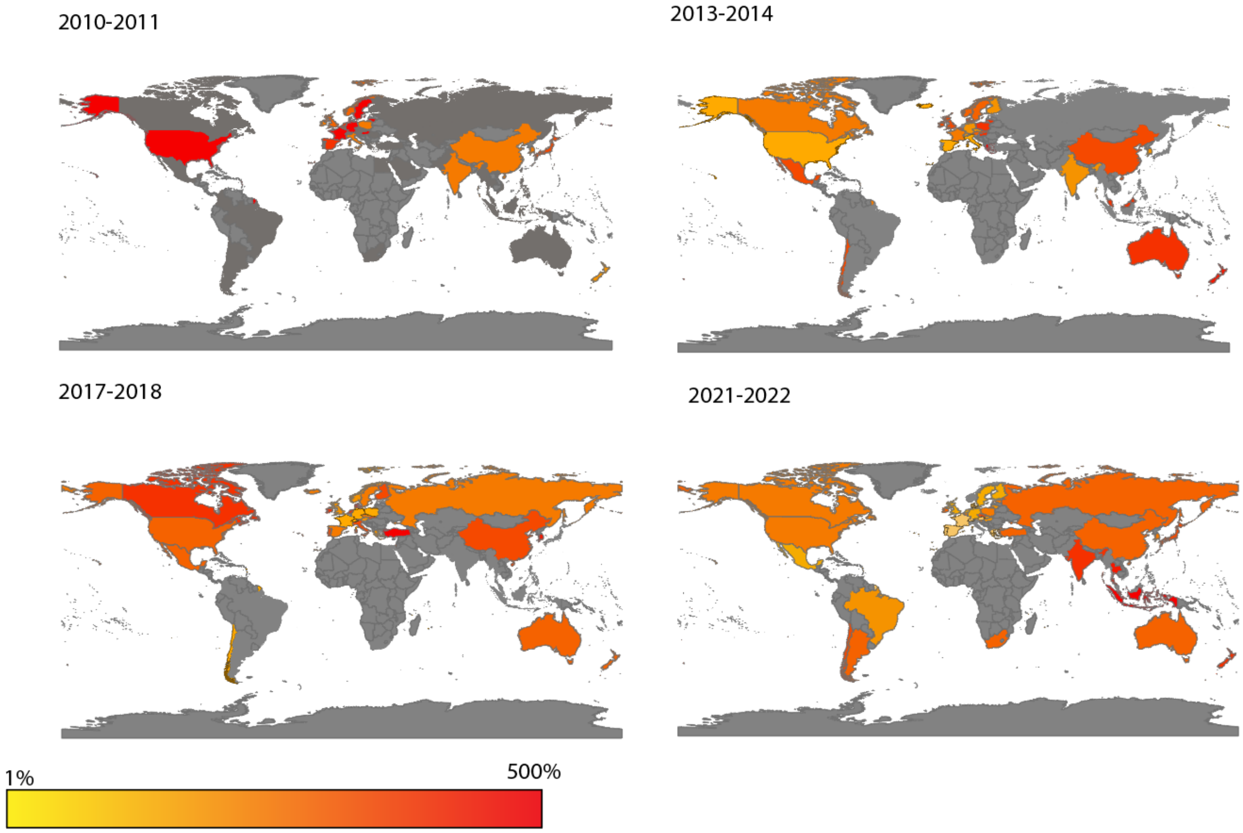

Suppl. Figure 9 | Growth of the electric vehicle (EV) market shares by country between 2010 and 2022. The growth of EV markets has been in the early 2010s most rapid in the lead markets of Europe, China and the US. However, this growth has in more recent years spilled out into vehicle markets of neighbouring countries. The base map image is the intellectual property of Esri and is used herein under license. Copyright © 2025 Esri and its licensors. All rights reserved.

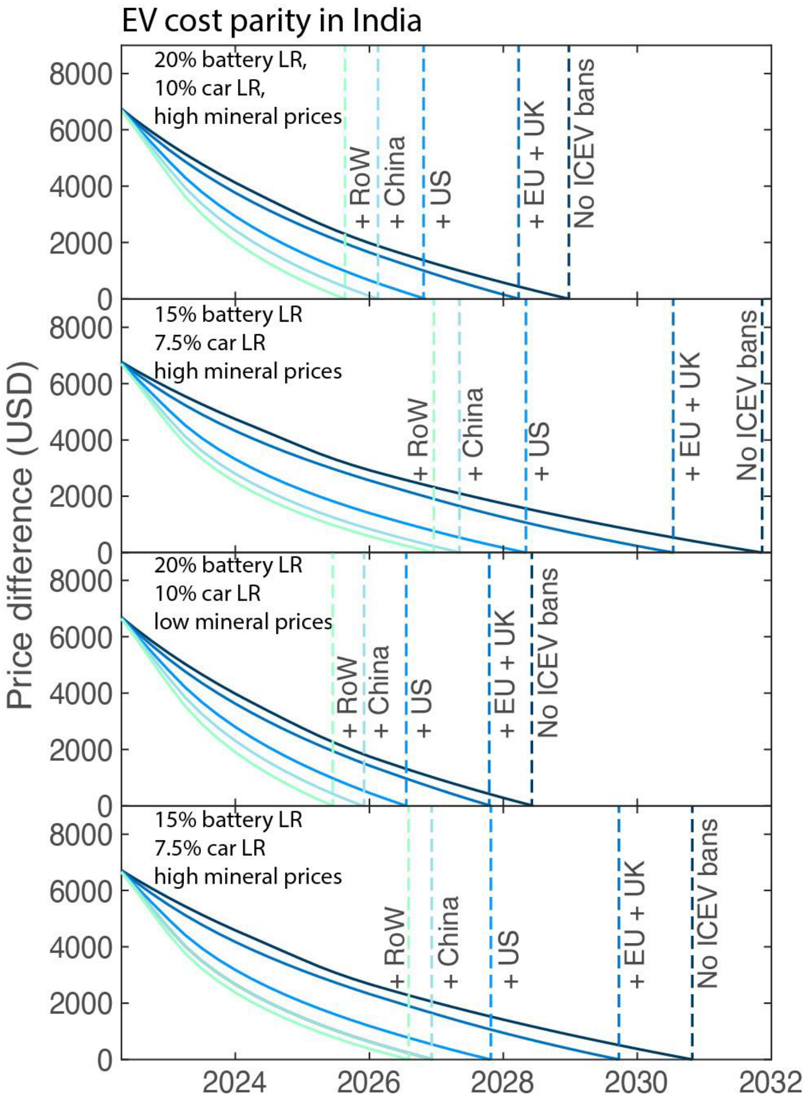

Suppl. Figure 10 | Trajectories for achieving cost parity for mid-range EVs in India. Scenarios and sensitivities include variations on assumed battery and vehicle experience curve rates as well as mineral prices. LR indicates learning rates, where car LR refers to the rate for cost reductions in the car excluding the battery, while battery LR is the rate that applies to the battery only. RoW stands for ‘Rest of the World’. EU stands for European Union, UK for United Kingdom, ICEV denotes Internal Combustion Engine Vehicle, while EV denotes electric vehicle.

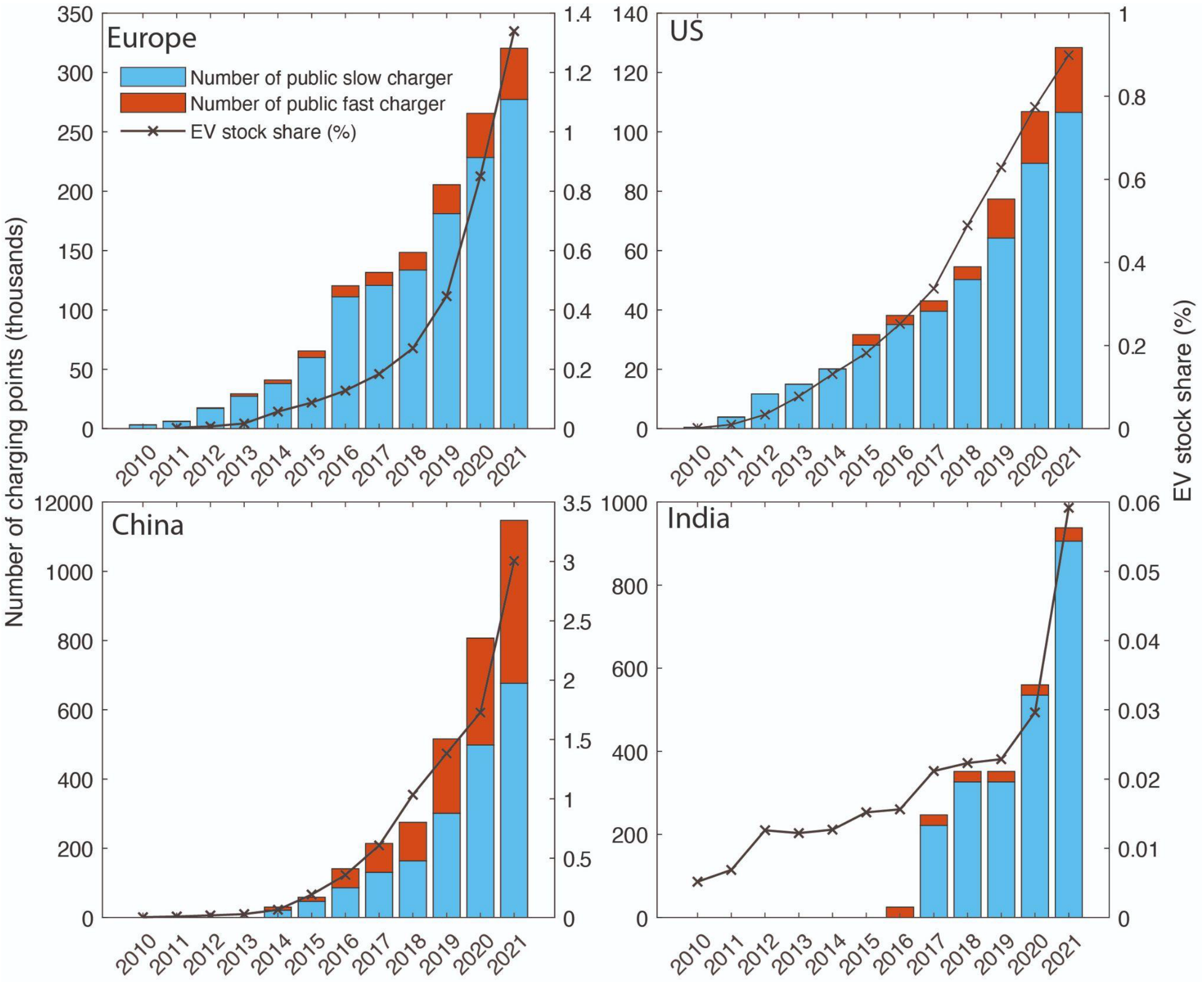

Suppl. Figure 11 | History of EV charging points. Historical diffusion of charging points (left axis and bars) and EV market shares (right axis and lines). EV denotes electric vehicle.

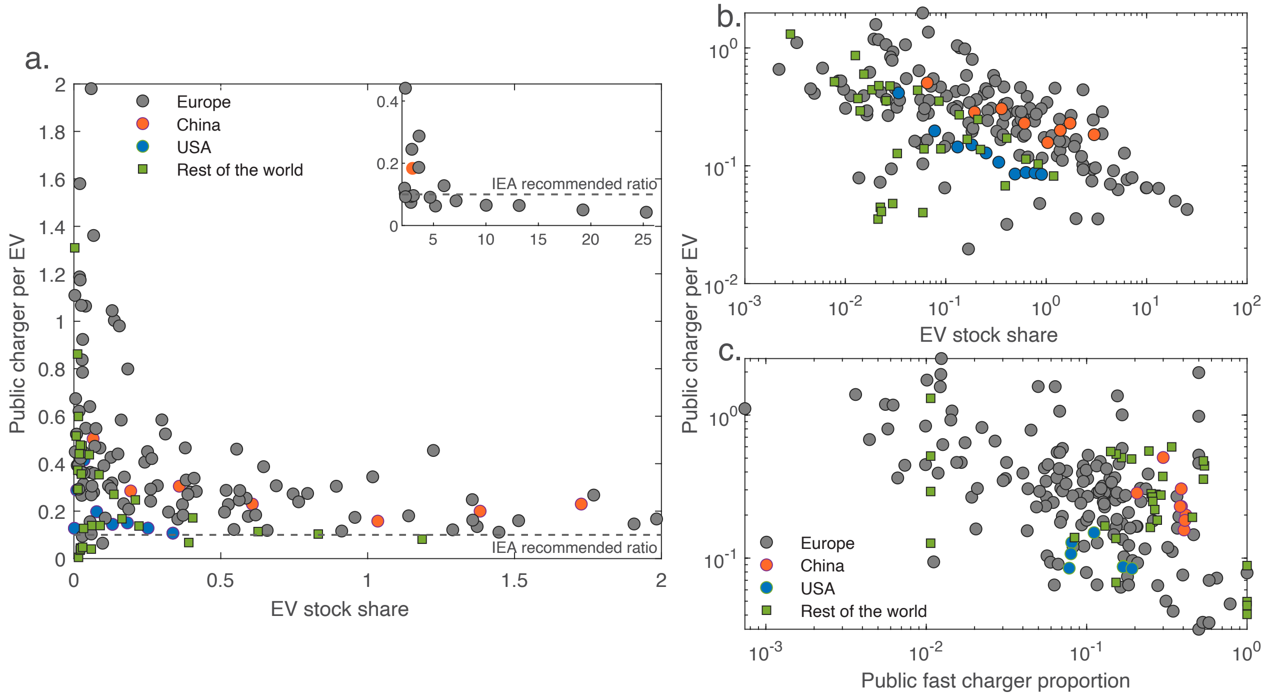

Suppl. Figure 12 | Charging point numbers per EV as EVs penetrate markets. a) Charging point per EV relative to the EV fleet size. The inset extends the horizontal axis. b) Same under log-log axes scaling. c) Charging point per EV as the proportion of public fast charger increases. EV denotes electric vehicle.

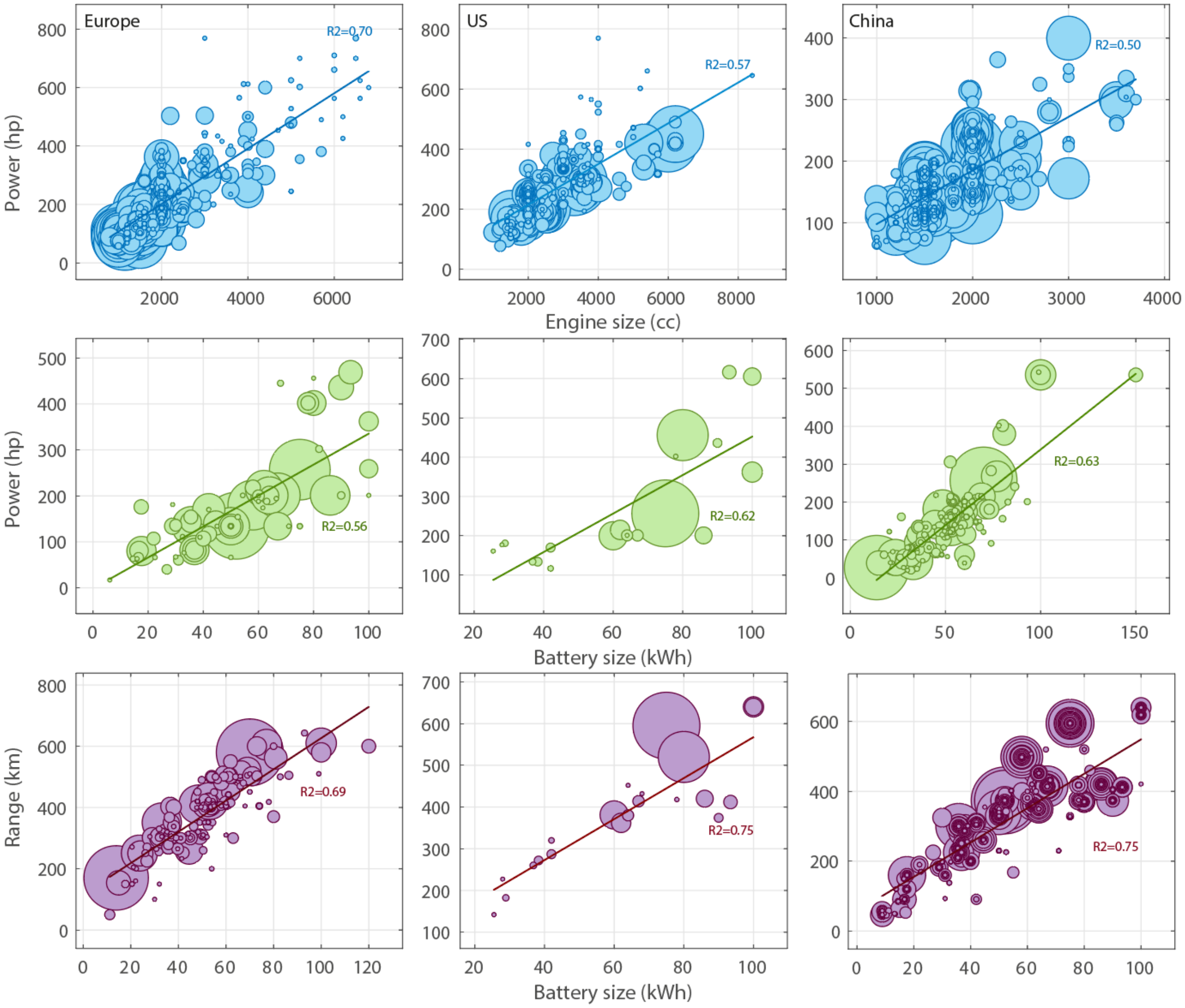

Suppl. Figure 13 | Properties of vehicles in the database. Sales weighted univariate regression between variables for Europe, the US and China. Each circle is shown per model, of which the area is scaled with the number of sales. First Row: power against engine size. Middle row: power against battery size. Bottom row: range against battery size.

Suppl. Table 1 | Vehicle database coverage and sources.

Variable	Region coverage	Time frame	Figures used	Source
**Vehicle sales by model**	Austria, Belgium, Bulgaria, Croatia, Republic of Cyprus, Czech Republic, Denmark, Estonia, Finland, France, Germany, Greece, Hungary, Ireland, Italy, Latvia, Lithuania, Luxembourg, Malta, Netherlands, Poland, Portugal, Romania, Slovakia, Slovenia, Spain, Sweden, UK, China, India, USA.	2016-2023	Fig. 3, Suppl. Fig. 5 Suppl. Fig. 6	Marklines
**Annual vehicle sales statistics by technology**	Australia, Austria, Belgium, Brazil, Canada, Chile, China, Denmark, Finland, France, Germany, Greece Iceland, Israel, India, Italy, Japan, Korea, Mexico, Netherlands, New Zealand, Norway, Poland, Portugal, South Africa, Spain, Sweden, Switzerland, Turkey, UK, US.	2010-2023	Fig. 2, Fig. 6, Suppl. Fig. 1, Suppl. Fig. 5 Suppl. Fig. 9	International Energy Agency
**Vehicle price and features by model**	France, Germany,, China, India, USA, Japan, Korea, Australia, Brazil, Canada, UK	2016-2023	Fig. 3, Suppl. Fig. 2 Suppl. Fig. 3 Suppl. Fig. 6 Suppl. Fig. 13	Marklines, Manufacturer websites

Suppl. Table 2 | The saturation levels, data sources, and values for α and β

Country	Saturation level (V*)	Data sources for V*	α	β
**Europe (average)**	550	Dargay et. al., 2007(10)	-3.07	-0.000138
**US**	800	Dargay et. al., 2007(10)	-17.85	-0.000207
**China**	300	Huo and Wang, 2012(61)	-2.05	-0.000735
**India**	400	Arora et. al., 2011(62)	-5.73	-0.000478

Suppl. Table 3 | Data sources

Main modules	Variable names	Data sources	Note
Vehicle technological profile	Suggested retail prices (MSRP)	Car manufacturers' websites	Collected from trusted car dealers and Electric Vehicle Database(24) if the MSRP is not available on car manufacturers' websites.
	Engine size		
	Battery size (PHEV/EV)		
	Fuel consumption		
	Power		
	Range (EV/PHEV)		
Model diversity	Number of models (by technology) available	MarkLines(25)	
	Car sales by model and technology		
EV manufacturing cost	Historical battery cost	Existing studies and leading car manufacturers (2, 26–35).	See Figure 1 and Suppl Figures 2-3.
	Learning rate	Existing studies (2, 28–30, 32, 35–38)	
	Historical rare metal costs	The World Bank (39), London Metal Exchange (40), mining.com (41)	See Suppl. Dataset for more information.
	Battery energy density improvement	BNEF(2); ICCT(42)	
EV pricing	Markup factor	UBS(29); ICCT(43)	
Cost of ownership	Fuel cost	The World Bank(44), GlobalPetrolPrices(45)	See Suppl. Dataset for more information.
	Electricity price	Eurostat(46); US EIA(47); GlobalPetrolPrices(48)	
	Maintenance cost (ICEV)	ICCT(42)	
	Maintenance cost (EV)	ICCT(42); BNEF(2)	
	Annual mileage	Odysse-Mure(49); China Statistical Yearbook(50); US highway Statistics(50)	
	Discount rate	Allcott and Wozny, 2014(51); Busse et al., 2013(52)	
Passenger travel demand projection	GDP per capita	The World Bank(53)	
	Road Length	Eurostat(54); Federal Highway Administration(55); China Statistical Yearbook(49)	
	Urbanization	The World Bank(56)	
	Urban density	The World Bank(57)	
	Historical average fuel economy standard	US BTS(58); ICCT 2018(59)	
Passenger fleet stock projection	Historical car stock	Eurostat (1); China Statistical Yearbook(49); India Ministry of Road Transport and Highways; Bureau of Transportation Statistics(60)	
	GDP per capita	The World Bank(53)	
	Car saturation level	Dargay and Gately(10, 11); Huo and Wang, 2011(61); Arora et. al., 2011(62)	

Notes: ICEV denotes Internal Combustion Engine Vehicle, PHEV denotes Plug-in Hybrid Electric Vehicle, and EV denotes electric vehicle.

Suppl. Table 4 | Regression results for personal road transport demand

Variable	OLS Coefficient	S.E.	t-stat	FE model Coefficient	S.E.	t-stat	Arellano-bond GMM Coefficient	S.E.	t-stat
PKM lag 1	0.56***	0.05	10.46				0.74***	0.06	12.30
Country	0.07***	0.01	6.22						
Ln(FP)	-0.15***	0.03	-4.55	-0.14***	0.05	-3.17	-0.08**	0.03	-2.46
Ln(U)	-0.01**	0.00	-2.42	-0.02***	0.00	-5.37	-0.01***	0.00	-2.93
Ln(Y)	0.03**	0.03	2.11	-0.01**	0.03	-2.03	-0.01**	0.02	-2.41
Ln(M)	0.02**	0.01	2.15	0.02	0.03	0.90	0.05**	0.02	2.28
Ln(UD)	-0.02***	0.03	5.20	-0.04***	0.22	6.69	-0.02**	0.19	2.56
Ln(FE)	-0.03***	0.01	-2.86	-0.02	0.14	-0.15	-0.14**	0.10	-2.09
Const	3.46***	0.01	-3.48	3.72	1.24	3.01	0.78	0.96	0.82
Bresch-Pagan test	1.58(0.21)								
Hausman test				35.02(0.00)					
Sargen test (P-value)							0.63		
N	166			166			158		
Adjusted R-squared	0.93			0.45					

**Means at the 5% significance level
***Means at the 1% significance level
Notes: PKM = distance driven per year in person kilometres, FP = fuel cost in terms of the oil price, U = urbanisation, M = road lengths, UD = urban density, FE = fuel economy of vehicles.

Suppl. Table 5 | Properties of vehicles by engine and battery size

Power (in hp)			
	Engine size ≤1400cc	1400cc<Engine size<2000cc	Engine size≥2000cc
Europe	119±61	218±144	329±61
US	170±7	215±37	450±319
China	123±61	196±120	257±140
**Power (in hp)**			
	Battery size ≤ 30kWh	30kWh<Battery size<70kWh	Battery size≥70kWh
Europe	100±80	155±150	301±167
US	105±10	166±50	400±202
China	90±71	119±61	172±190
**Range (km)**			
	Battery size ≤ 30kWh	30kWh<Battery size<70kWh	Battery size≥70kWh
Europe	177±120	350±200	506±136
US	184±43	362±90	507±133
China	185±140	129±138	435±205

Supplementary Note 3. Defining a tipping point in socio-economic systems

The definitions for tipping points in strictly defined dynamical mathematical systems, and in some cases in the earth system, are largely well established and agreed.[28–30] Variables monitored for tipping points will typically involve clearly defined quantities or measured physical quantities that are also represented, for instance, in general circulation climate models.

For socio-economic systems, tipping points have been studied but definitions vary and are not established with any consensus, and suffer from some degree of ambiguity in terms of measurable variables.[31–33] Furthermore, variables undergoing tipping points may in some cases not be measurable, as they involve perceptions and attitudes of persons. That is partly because theories describing human behaviour are fluid and subject to some degree of interpretation, change according to context or are difficult to validate empirically to the degree that can be done in physical systems. It is thus useful, for the present work, to elaborate regarding the definition that we adopt for a socio-economic tipping point.

The most important characteristic of the EV tipping point for which we present evidence here, we argue, is the condition of positive feedback, where the evolution of the system becomes self-propelling towards the new state of EVs dominating markets. The threshold for exactly what 'dominance' means is unimportant if the forces acting on the system pull it in the direction of that new state. Thus positive feedbacks in the movement towards EV dominance is critical, where movement induces a pulling force reinforcing that movement.

This does not strictly mean that reaching the new state of EV dominance becomes inevitable, as many drastic changes of context could still disrupt the system's movement, such as a complete reversal of policies, or a large economic shock. Thus we cannot rule out that a reversal of policy could stall or reverse the diffusion of EVs. It does indicate however that conditional to no drastic change in context taking place (e.g. no change in policy regime), the system accelerates its movement towards the new state of EV dominance.

It is thus important to define the nature and source of positive feedback. In equilibrium systems, an attractor for the dynamical system described is postulated to exist and to be stable. This equilibrium point moves in state space according to socio-economic variables, and maintains the position of the system within that stable state. Fluctuations pushing the system out of equilibrium are ultimately overcome by the force bringing the system back towards its equilibrium point. In this case, this would concern an equilibrium composition of the vehicle fleet that depends on prices, incomes and other socio-economic parameters.

However, technology diffusion systems are typically not in equilibrium[7,12], because the decision to adopt a technology depends on the information that agents have about those technologies, and agents acquire that information via observation of technology use. This creates a nonlinear positive feedback in which the diffusion of a technology facilitates its own further diffusion[34,35]. However, the diffusion process is slow, as technology use and production takes considerable time on the scale of economic evolution. Such a positive feedback precludes the existence of a stable equilibrium under the timescale of economic measurement (years).

Positive feedbacks (and increasing returns to scale) in technology systems stem from the innovation process, in which once new knowledge or industrial capabilities are created, they are typically not forgotten or abandoned. Thus the diffusion of innovations not only self-propels via the diffusion process, but also, via gradually eliminating cost hurdles impeding its own diffusion, leading to an acceleration[4].

In the FTT model, we proxy the positive feedback using the measured cost and price relationship with cumulative production (Suppl. Note 1), coupled with the price relationship with the technology adoption process (Suppl. Note 2). As the cost differential between EVs and ICEVs declines and

reverses to become negative, and as the growth of EVs in the marketplace attract further growth, each element of diffusion increases the force acting on agents inducing them to adopt EVs further.

Current policies supporting the diffusion of EVs generally aim to induce sufficient diffusion to achieve price parity. Under our definition and model, exceeding real price parity between EVs and ICEVs (excluding subsidies or taxes) mostly confers the advantage that with no further policy support, the diffusion of EVs can be expected to continue towards gradual dominance. Hence we use the moment of price parity as a critical measurable milestone for self-propelling diffusion.

Supplementary Note 4. Assumptions concerning contextual factors

FTT:Transport projections are not pure forecasts, they are forecasts conditional to a number of assumptions remaining true over the projection period. If any of these assumptions are broken, the forecasts cease to be valid. Here we discuss the most important contextual factors that must remain true for the projections to maintain their validity.

The first assumption implicit in any FTT projections are that existing policies remain in place, as a backdrop upon which new policies are added. This is implicit in the definition of the 'intangibles' parameter γ_i, which measures all unknown cost factors not covered by the LCOT metric in order to reproduce observed diffusion rates seen in the historical data. Most importantly, some policy instruments may not be expressed explicitly in the policy database of FTT:Transport, and therefore are covered implicitly via γ_i. Our assumption that γ_i remains constant over the forecast implies that existing policies are not revoked.

The second important assumption in our modelling concerns economic and preference parameters for which we have no information that could justify to impose any substantial changes over the forecast period. This includes the consumer discount rate, interest rates, exchange rates, rates of economic growth, trade patterns, social order, and political dynamics. Substantial changes in these parameters may invalidate FTT forecasts.

Lastly, FTT:Transport assumes that resources are available to manufacture new vehicles in each technology category in sufficient numbers to cover the emergent demand. In the Methods, we discuss our analysis of the availability of critical materials needed to manufacture EVs. A wider range of materials are also required to be available at prices at which the vehicles can be manufactured at competitive costs. As discussed in the Methods, in all plausible scenarios of critical material prices, vehicle price parity continues to be achieved, showing robustness in our results.

A critical implication of the above is that although tipping points are defined by their positive feedbacks, this remains conditional to the above contextual assumptions remaining valid over the projection period. A tipping point could in principle be averted via substantial changes in context, notably a radical policy reversal (e.g. banning EVs).

Supplementary Note 5. The role of model variety

The role of model variety is important in the transition towards EVs, as it determines the price difference between an EV and a comparable ICEV. Suppl. Figure 14 illustrates the impact of model diversity on the subsidy required. In markets where very few EV/PHEV models are available, only a few consumers can find a low-carbon equivalent of the ICEVs. To convince consumers to switch to EVs in markets where their variety is low, the subsidies that could break even on a cost basis must bridge potentially wide gaps between the prices of some conventional vehicle market segments and those of scarce zero-carbon alternatives. However, where variety is as large for EVs as for ICEVs, break-even subsidies can be relatively low, as they are only required to bridge the average price difference between EVs and their corresponding conventional counterparts.

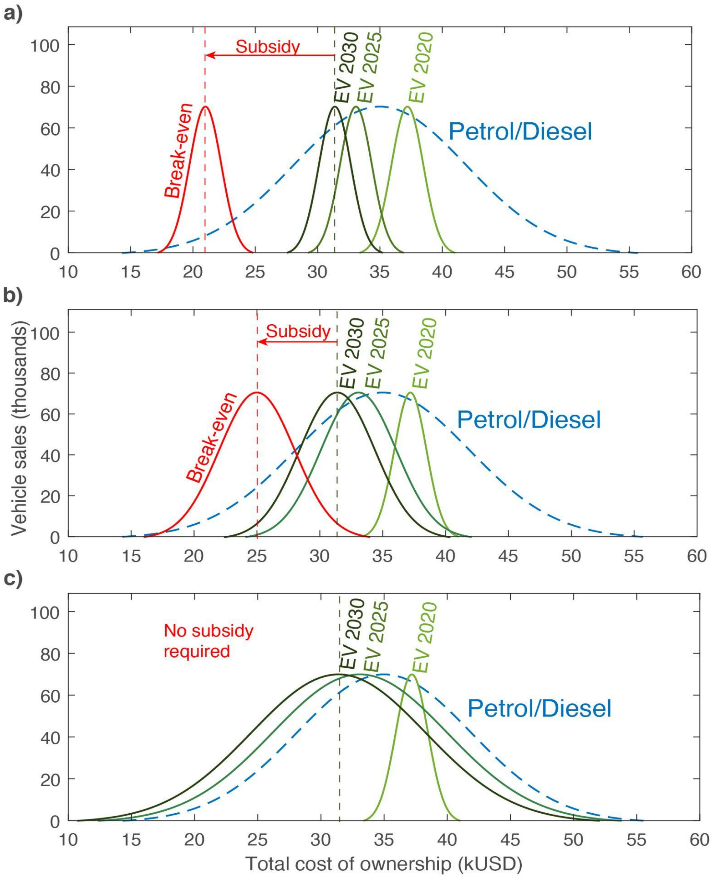

Suppl. Figure 14 | Relationship between model variety and the breakeven subsidy. The breakeven subsidy declines as costs decline with rising investment, for (a) constant EV variety, (b) doubled EV variety and (c) EV variety matching the current variety of petrol/diesel vehicles. EV denotes electric vehicle.

Supplementary Note 6. Estimating abatement costs and the impacts of tariffs

To inform public policy, it is useful to estimate the welfare costs of technological change scenarios. FTT:Transport does not optimise its systemic cost, and nor does the car fleet in the real world, as our data clearly shows. Vehicles are, as appears from the data, chosen more on the basis of their popularity, price and perceived value, than on the basis of what minimises whole system cost (see [13]). Our dataset however also shows that the EV transition nonetheless cuts the costs of owning and operating a vehicle, to the extent that in all regions studied, cost parity with conventional vehicles occurs. We show in Suppl. Figure 15 how different policy instruments interact in FTT, where some policy mixes generate synergies while others interfere. We show in Suppl. Figure 16 the abatement cost, in $/tCO2, associated with scenario (d) of Figure 5. This is calculated as the difference in cost per vehicle-km in each vehicle class range between EVs and all types of fossil fuelled vehicles, divided by the avoided emissions (in gCO2/km), and averaged over the fleet. The initial trajectories (for each market) cross cost parity, which implies negative abatement costs, in around 2025-2027 for the EU, US, India, and in the past for China. However, the absolute negative abatement costs gradually return towards zero going to 2050. The reason is that with increasing disposable income released by cost reductions, some agents use this to purchase larger and more luxurious EVs (e.g. sports-utility vehicles, larger batteries), pushing up the average (in the context of rising model variety). The side-effect of this is to partially absorb the green discount in abatement cost observed in the early years due to experience curves.

The recent imposition of trade tariffs could however change this picture. Given the rapidly changing landscape of trade policy between the main EV producers, it is natural to ask whether those changes could stop or reverse the occurrence of EV tipping points. This is a complex question since (1) all three lead markets largely have the industrial capabilities needed to produce EVs entirely domestically, which could therefore avoid tariffs; (2) however capacities to produce batteries in the EU and US does not currently keep up with EV sales, the balance in the battery market originating from China. Thus in the short term, a scenario of tariffs on EVs and/or batteries, imposed bilaterally between all three lead markets, could affect EV markets in the EU and the US, but probably not in China, given that China is the major exporter of batteries. In the long run, under high tariffs, the US and EU would likely expand their productive capacities to avoid any imports, but in the short run, tariff costs would likely be partially passed on into EV prices, and affect sales.

We explore this by assuming tariffs on batteries only in the EU and US, as these are the sensitive vehicle components that would be affected, while other vehicle components can be produced domestically and avoid tariffs. In Suppl. Figure 17, we explore the impacts of 50% and 100% tariffs on batteries passed-on to consumer prices and measure the change in year of cost parity. We see that cost parity could be delayed by up to 10 years. In these scenarios, the diffusion of EVs could therefore be affected in the EU, US, but not China. This could also delay the uptake of EVs in the rest of the World as a result of slower cost reductions. However, this timescale is longer than the time it would take to scale up battery productive capacity, and while there could be delays, we find no evidence that tipping points could be prevented by tariffs from occurring.

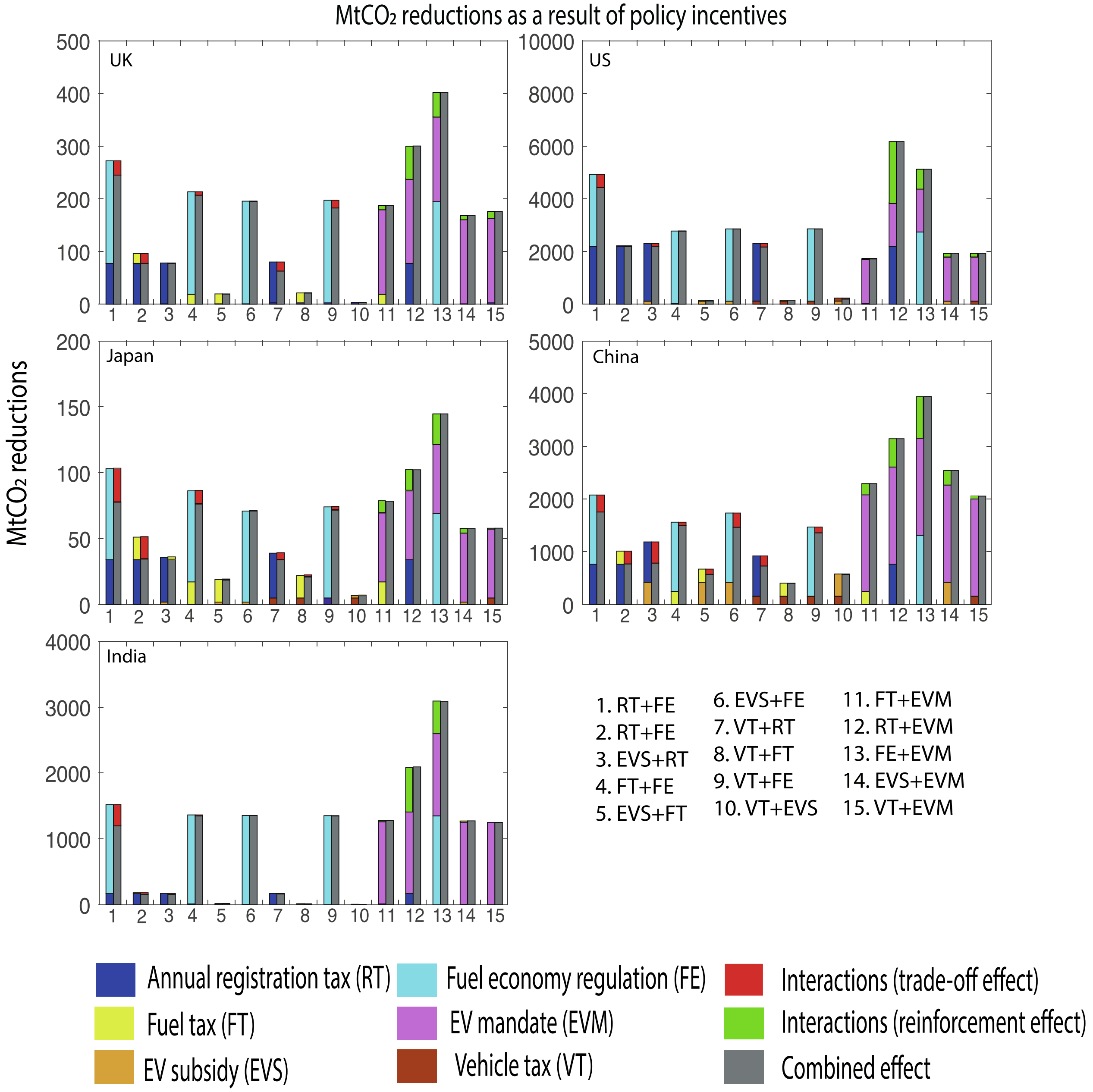

Suppl. Figure 15 | Interactions between policy instruments in FTT:Transport. The impact of the combination of policies can be more (reinforcement) or less (interference) than the sum of their effects when taken in isolation. Reproduced from Lam & Mercure[10].

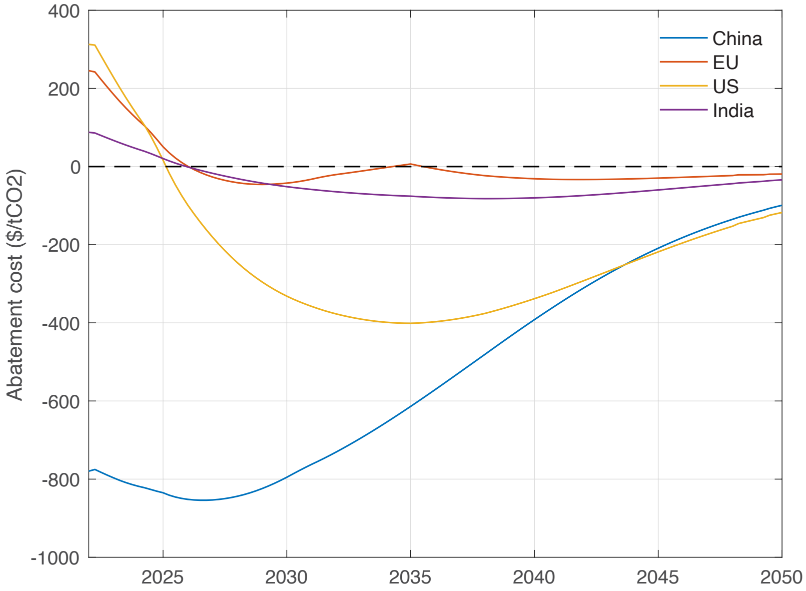

Suppl. Figure 16 | Abatement costs associated with scenario (d) of Figure 5. Cost differences between EVs and ICEVs per km, divided by the avoided emissions per km for each ICEV type, averaged over all technologies and vehicle power classes. Cost declines are due to learning curves, whereas cost increases are due to agents upgrading to more expensive and more powerful vehicles. ICEV denotes Internal Combustion Engine Vehicle, while EV denotes electric vehicle.

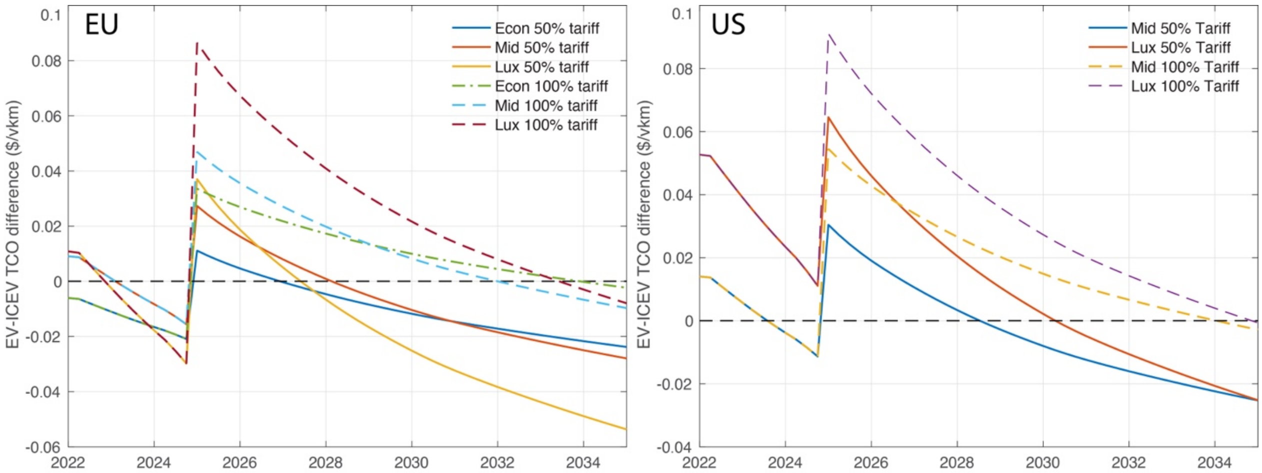

Suppl. Figure 17 | Impacts of tariffs. Impacts of the imposition of tariffs on battery imports for the cost of vehicles that use imported batteries. The sudden imposition of tariffs on batteries (50% in solid lines and 100% in dashed lines) sets back reaching cost parity by up to 9-10 years, but it remains achieved eventually in all cases. 'Econ' stands for economic, 'Mid' for mid-range and 'Lux' for luxury, which refer to the engine/motor-battery size class.

Supplementary Note 7. Sensitivity analyses

It is important to understand the sensitivity of EV price and cost parity moments to key parameters. Sensitivity analyses were carried out to examine the impact of key parametric uncertainties on the lifetime cost of ownership and on the scenarios under the current trajectory scenario and the policy combination scenario (current trajectory + taxes + subsidies + regulations + EV mandates, see Methods and Suppl. Table 6 for policy assumptions).

As a starting point, a comparison of our FTT:Transport projections is made with International Energy Agency projections, shown in Suppl. Figure 18.

The lifetime cost of ownership for EVs depends on ten categories of parameters that impact EV prices and the costs of owning EVs. The parameters tested include: i) battery material costs, ii) battery learning rates, ii) battery energy density improvement rates, iii) EV price markup factors, iv) tax and subsidies, v) consumer discount rates, vi) oil/electricity prices, vii) EV/ICEV maintenance costs, viii) perceived costs (γ values, see Suppl. Note 2), ix) annual distance, x) battery driving range. Changes in the input parameters are not mutually exclusive, though some combinations are more likely than others. For example, prices for several raw materials have been surging recently. Vehicle maintenance costs and oil/electricity prices can go up or down simultaneously. Hence, we tested the combined effect of material cost volatility and uncertainties in ownership costs.

The size of the variations is justified based on existing literature or a range we consider reasonable (see Suppl. Table 7). The variations of the parameters and the impact of the variations are shown in Suppl. Tables 8-15. We are interested in the changes in ownership costs and EV adoption (i.e. market share) to variations in key parameters. The resulting changes are reported in percentage changes from the scenario without variations. As a benchmark, we adopt the definition that a change of X% in total cost of ownership (TCO) or market shares that result from a parameter variation of Y% is 'large' if X is of the order of or greater than Y, and 'not large' if X is much smaller than Y. This is because if X% is larger than Y% parameter variation, we may see large propagating uncertainty in the model, otherwise not.

We observe that material cost variations have only a relatively small impact on the changes in EV TCO. This is consistent with the analysis of BNEF (2021)[36] that a doubling of the prices of one of the key rare metals for EV (e.g. cobalt, lithium and nickel) would increase the battery costs by less than 10%. If the material costs are reduced by 50%, the battery cost would drop by less than 9%. Among the three key metals for batteries, the volatility of Cobalt and Nickel costs has a larger impact on the battery pack cost than the cost of Lithium. Even if the prices of cobalt, lithium and nickel all double, the battery pack price only increase by less than 20%. Therefore, by implementing these measures collectively, variations in material costs would only increase the TCO by less than 2.1% in all regions and result in a less than 5% change in the share of EV in the current trajectory scenario. As EVs gain market shares, the impact of material costs on the TCO and EV/PHEV shares are smaller in the 'strong policies scenario' than in the current trajectory scenario.

There are many possible pathways through which battery improvements can lead to battery price declines or increases. We analysed the impacts of learning rates, energy density and driving range uncertainties on the TCO and EV adoption. Overall, these variables tend to have a small impact on the TCO and change in technological shares, i.e. less than a 6% change in TCO result from reasonable variations in these variables. Among these variables, variations in the learning rates have relatively larger effects on TCO than the driving range and the improvement in battery energy density. The effects of the variations in battery improvement rates on TCO are larger in countries/regions with a low EV price (i.e. China) than in countries with a relatively high EV price (i.e. Europe). For example, a 50% change in the battery learning rate (i.e. going from 20% to 10%

or 30%) results in about a 15% difference in the cost of the battery in 2035, and around a 5% change in TCO in China and a 3% difference in Europe in that year.

EV price fluctuations lead to more significant variations in TCO in comparison to battery price fluctuations. Other than the battery price fluctuations, car prices can be impacted by the perceived costs, EV incentives (including taxes/subsidies) and markup factors. EV incentives such as subsidies can directly impact the EV price competitiveness, leading to more than a 20% change in EV shares in the US and China, in the current trajectory scenario. Over time, car manufacturers may increase markup factors for EVs gradually, as EV performance and price advantages improve. In a scenario where car manufacturers increase markup factors by 20%, car prices can increase by around 20% in all four regions in the current trajectory scenario. However, it is unlikely that car manufacturers and dealers would rapidly push up markup factors before EVs have gained a price advantage over petrol cars. In all our policy scenarios where EVs gain significant shares as a result of a policy package, variations in the markup factors have a very small impact on the change in EV shares. For all four regions, a 20% increase in the markup factors would only lead to less than a 1% change in EV shares. Hence, when market shares for EVs are high, an increase in markup factors and car prices have a negligible impact on EV adoption.

The impact of ownership cost component variations on the overall TCO is relatively small in all cases. By varying the consumer discount rate by 10%, the TCO could change from 4%-8% and shift the price parity year by 1-2 years in both the baseline and the ‘strong policies’ scenarios. Among the cost factors that add up over the lifetime of a car, fluctuations in oil prices have a relatively larger effect on ICEV ownership than other cost factors. For example, a surge in oil prices by 50% leads to an increase in TCO by 4% to 6%. This could shift the price parity years up by 1 year and increase the percentage change of EV/PHEV shares by 5% to 9% in the baseline scenario. Uncertainties in other cost factors, such as the EV/ICEV maintenance costs have minor effects on the change in TCO and in the change in the share of technology. If variations in the discount rates and the cost factors took place simultaneously, the TCO for ICEVs can change by up to 15% and by up to 5% for EVs. In the current trajectory scenario, this can alter the change in technology shares in 2035 by up to around 10%. The uncertainties in the ownership costs have a minor effect on the adoption of EVs in the ‘strong policies scenario’, with the percentage change in EV shares being less than 1% when all cost factor uncertainties are combined.

Lastly, we analyse the sensitivity of scenarios to changes in stringency of policy assumptions. Suppl. Table 6 provides the absolute policy stringencies for each country and instrument used. Note that subsidies are not assumed in China, and that taxes are additional to existing taxes in all regions. In Suppl. Tables 16-19, we vary taxes, subsidies and mandates by 10%, and change the year of ICEV phaseout by 2 years. The outcomes are measured in terms of relative changes in market shares in 2050 against the undisturbed scenario. The model is most sensitive to changes in mandates and changes in the year of ICEV phaseout, but the outcomes do not materially change the policy conclusions. There are no optimal solutions, it would be up to policy-makers to balance stringency against other political considerations.

Policies also interact with one another, as we discuss in Figure 5 of the main paper. In Suppl. Figure 15, we show in more detail how policy instruments interact in FTT:Transport, reproduced from earlier work that focused specifically on that.[10] We find that some pairs of instruments cause total emissions reductions greater than the sum of their impacts when used individually, hence they synergise, whereas other pairs of instruments interfere. These considerations have guided our choices of policy instruments in the present work.

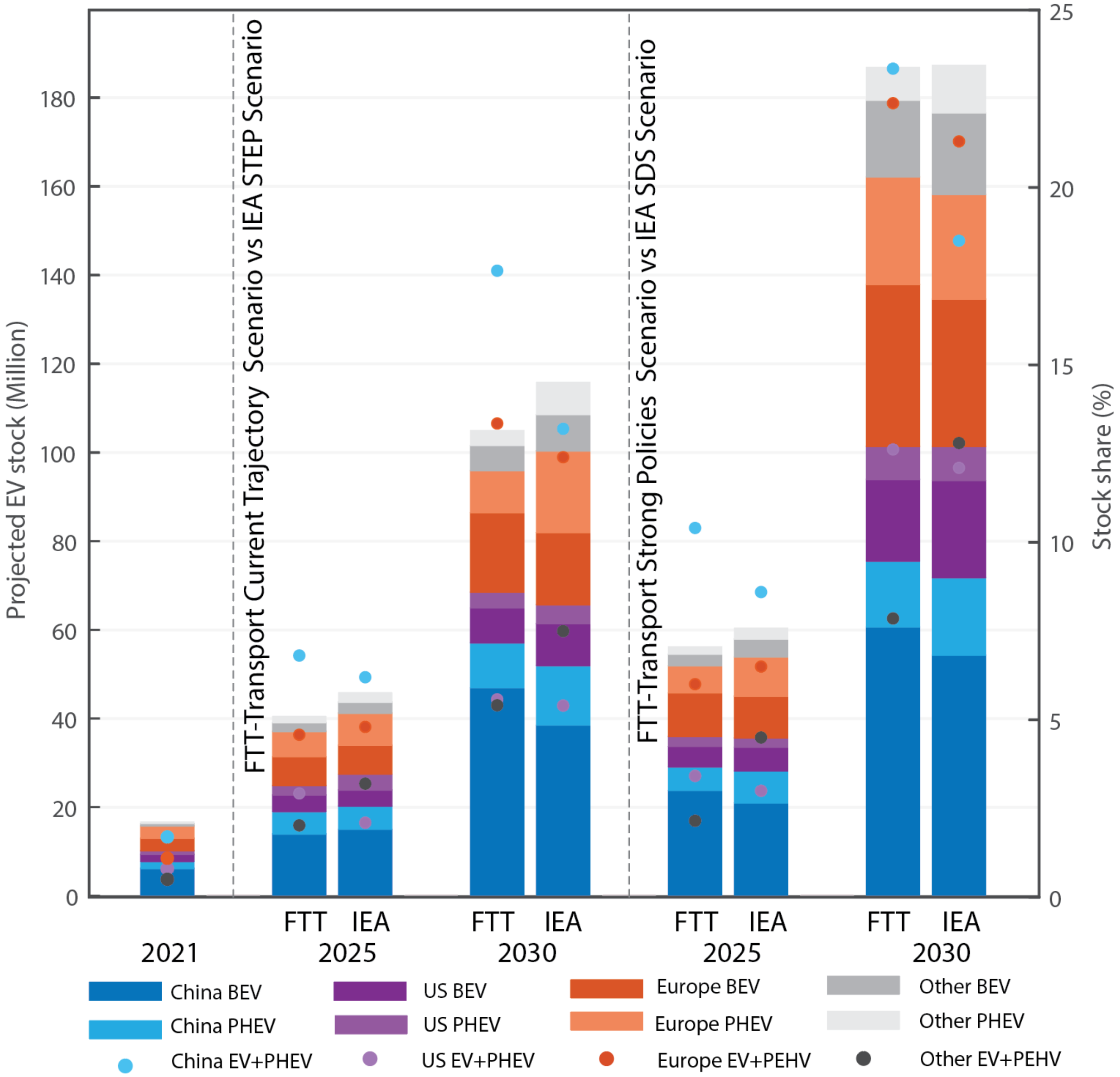

Suppl. Figure 18 | Comparison of projected EV stocks between our model FTT and the IEA. Comparison between the current trajectory scenario and the policy combination scenario presented in this paper, alongside International Energy Agency (IEA) projections in the Stated Policies Scenario (STEPS) and Sustainable Development Scenario (SDS) from the IEA's EV database. The bars are the projected EV stocks and the dots are the projected EV stock shares. ICEV denotes Internal Combustion Engine Vehicle, while EV denotes electric vehicle.

Suppl Table 6 | Stringency of policy instruments in FTT modelling (Fig. 5 scenario d)

		2025	2030	2035
**EV Mandate US**		15%	30%	Not assumed
**EV Mandate EU**		25%	40%	Not assumed
**EV Mandate China**		30%	60%	Not assumed
**EV Mandate India**		6%	25%	Not assumed
**ICEV phaseout US**		-	-	In place
**ICEV phaseout EU**		-	-	In place
**ICEV phaseout China**		-	-	In place
**ICEV phaseout India**		-	-	In place
**ICEV tax US**	**Econ**	$3,000	$3,000	$0
	**Mid**	$5,000	$5,000	$0
	**Lux**	$7,000	$7,000	$0
**ICEV tax EU**	**Econ**	$1,183	$1,183	$0
	**Mid**	$2,406	$2,406	$0
	**Lux**	$6,010	$6,010	$0
**ICEV tax China**	**Econ**	$2,000	$2,000	$0
	**Mid**	$3,750	$3,750	$0
	**Lux**	$7,300	$7,300	$0
**ICEV tax India**	**Econ**	$520	$520	$0
	**Mid**	$1,200	$1,200	$0
	**Lux**	$1,800	$1,800	$0
**EV subsidy US**	**Econ**	$5,000	$5,000	$0
	**Mid**	$10,000	$10,000	$0
	**Lux**	$15,000	$15,000	$0
**EV subsidy EU**	**Econ**	$4,400	$4,400	$0
	**Mid**	$6,000	$6,000	$0
	**Lux**	$9,000	$9,000	$0
**EV subsidy China**	**Econ**	$0	$0	$0
	**Mid**	$0	$0	$0
	**Lux**	$0	$0	$0
**EV subsidy India**	**Econ**	$8,000	$8,000	$0
	**Mid**	$12,000	$12,000	$0
	**Lux**	$16,000	$16,000	$0

Notes: ICEV denotes Internal Combustion Engine Vehicle, PHEV denotes Plug-in Hybrid Electric Vehicle, and EV denotes electric vehicle, ‘Econ’ stands for economic, ‘Mid’ for mid-range and ‘Lux’ for luxury, which refer to the engine/motor-battery size class.

Suppl. Table 7 | Justification for the variations for the sensitivity analyses.

Parameter	Range	Justification	Note
**Lithium cost**	±50%,+100%	Market prices for Lithium between 2000-2021[38]	We assumed that price volatility larger than 100% will be mitigated through increasing mining capacity over time.
**Cobalt cost**	±50%,+100%	Market prices for Cobalt between 2000-2021[38]	
**Nickel cost**	±50%,+100%	Market prices for Nickel between 2000-2021[38]	
**Learning rates**	±10 percentage points	Learning rate variations were considered based on existing studies[36,39–46]	Learning rates used are between 10% and 30% with a mean of 20%.
**Battery energy density**	±3%	Average battery density improves at 7% annually over the past ten years[36]	Battery energy density improvements are between 1% and 7% with a mean of 4%.
**Driving range**	±50%	Gradual increase in driving range, up to 50% higher than the baseline level in 2035, consistent with assumptions in the BNEF, 2021[43] and findings in Nykvist et. al., 2019[43]	
**Perceived costs**	±20%	20% is the maximum systematic error on the mean capital cost we consider possible at one STD.	Varying the perceived cost (γ value) above 20% would violate the model because the diffusion trajectory will no longer be consistent with the historical trends.
**Markup factor**	+30 percentage points	We assumed that the markup factor for EVs increases gradually to eventually reach 'profit parity' with petrol cars. We consider a markup factor of 30% to be a reasonable upper limit by 2035 based on the analysis of ICCT, 2021[47] and the findings in Velzen et. al., 2019[48].	The markup factor in the current trajectory scenario is 10%.
**EV price**	±20%	Depending on the sources of EV price uncertainty, 20% of the car price is considered a reasonable maximum variation between different variants of an EV model.	The main sources of EV price uncertainty are model updates, model variants and changes in policy incentives.
**Discount rate**	±10 percentage points	Following Allcott and Wozny, 2014[49] and Busse et al., 2013[50], we test a low consumer discount rate scenario (10%) and a high consumer discount rate scenario (30%).	The discount rates used are between 10%-30% with a mean of 20%.
**Oil price**	±20%, ±50%	Crude oil prices fluctuations between 2000 and 2021[51]	The fuel price assumptions in the current trajectory scenario are shown in the Suppl. Dataset. We assumed that oil price volatility larger than 50% than the baseline level of a short term shock.
**Electricity price**	±50%	Electricity prices fluctuations between 2000 and 2021[52]	The household electricity price in the current trajectory scenario for individual regions is shown in the Suppl. Dataset.
**EV maintenance cost**	±20%	Based on the reported ranges[47], we assumed 20% is the maximum maintenance cost error.	The baseline maintenance assumptions for EVs are shown in the Suppl. Dataset.
**ICEV maintenance cost**	±20%	Based on the reported ranges[47], we assumed 20% is the maximum maintenance cost error.	The baseline maintenance assumptions for ICEVs are shown in Suppl. Dataset.
**Annual distance**	±20%	Depending on fuel type, following official statistics on vehicle annual mileage between 2010 and 2020[53–55], we test a low average annual distance scenario (12000 km) and a high annual distance per year scenario (18000km).	

Notes: ICEV denotes Internal Combustion Engine Vehicle, PHEV denotes Plug-in Hybrid Electric Vehicle, and EV denotes electric vehicle

Suppl. Table 8 | Sensitivity analyses for the current trajectory scenario for Europe.

Sensitivity parameters			Var	% Change in Total Cost of Ownership (2035)					%Change in technology shares in 2035				
				Petrol	Diesel	Hybrid	PHEV	EV	Petrol	Diesel	Hybrid	PHEV	EV
Current trajectory scenario	Europe	**Material cost**											
		Lithium cost	-50%	0.00	0.00	0.00	-0.04	-0.21	-0.03	-0.02	-0.55	0.05	0.23
		Lithium cost	+50%	0.00	0.00	0.00	0.01	0.03	0.00	0.00	0.01	-0.01	-0.03
		Lithium cost	+100%	0.00	0.00	0.00	0.05	0.24	0.03	0.03	0.06	-0.04	0.29
		Cobalt cost	-50%	0.00	0.00	0.00	-0.08	-0.38	-0.04	-0.04	-0.09	0.07	0.51
		Cobalt cost	+50%	0.00	0.00	0.00	0.02	0.13	0.01	0.01	0.02	-0.01	-0.12
		Cobalt cost	+100%	0.00	0.00	0.00	0.06	0.33	0.01	0.03	0.07	-0.06	-0.40
		Nickel cost	-50%	0.00	0.00	0.00	-0.09	-0.40	-0.05	-0.05	-0.11	0.08	0.53
		Nickel cost	+50%	0.00	0.00	0.00	0.02	0.14	0.01	0.01	0.03	-0.01	-0.13
		Nickel cost	+100%	0.00	0.00	0.00	0.07	0.36	0.01	0.04	0.08	-0.07	-0.42
		Combined cost	-50%	0.00	0.00	0.00	-0.20	-1.09	-0.13	-0.11	-0.25	0.20	1.27
		Combined cost	+50%	0.00	0.00	0.00	0.07	0.27	0.05	0.05	0.08	-0.05	0.36
		Combined cost	+100%	0.00	0.00	0.00	0.20	1.06	0.13	0.11	0.25	-0.19	-1.27
		**Battery improvement**											
		Learning rates	-10%	0.00	0.00	0.00	0.52	2.77	0.24	0.20	0.47	-0.35	-2.30
		Learning rates	+10%	0.00	0.00	0.00	-0.34	-1.81	-0.17	-0.15	-0.34	0.27	1.68
		Battery energy density	+3%	0.00	0.00	0.00	-0.17	-0.92	-0.07	-0.06	-0.14	0.11	0.67
		Battery energy density	-3%	0.00	0.00	0.00	0.40	2.13	0.14	0.12	0.28	-0.20	-1.32
		Driving range	+50%	0.00	0.00	0.00	0.47	2.50	0.15	0.13	0.30	-0.21	-1.46
		Driving range	-50%	0.00	0.00	0.00	-0.29	-1.54	-0.11	-0.09	-0.22	0.16	1.05
		**EV price**											
		Perceived costs	+20%	0.00	0.00	0.00	10.47	12.43	1.55	1.36	2.91	-11.30	-10.54
		Perceived costs	-20%	0.00	0.00	0.00	-9.11	-11.82	7.23	-10.31	-14.84	7.26	6.65
		Markup factor	+10%	0.00	0.00	0.00	8.70	9.08	0.77	0.66	1.56	-4.35	-3.86
		Markup factor	+20%	0.00	0.00	0.00	17.38	18.17	1.58	1.35	3.20	-9.90	-8.10
		EV price	+20%	0.00	0.00	0.00	0.00	20.22	2.02	1.72	4.11	4.00	-18.20
		EV price	-20%	0.00	0.00	0.00	0.00	-21.54	-1.14	-1.00	-2.16	-3.10	20.79
		**Ownership cost**											
		Discount rate	+10%	-6.10	-4.86	-7.15	-5.39	-2.54	3.66	-4.57	3.04	1.77	-1.09
		Discount rate	-10%	5.21	6.21	7.21	6.21	4.10	-7.16	6.97	-0.90	-0.10	2.70
		Oil price	+20%	2.95	1.98	3.21	1.87	0.00	-4.04	2.70	0.31	4.55	2.45
		Oil price	+50%	5.89	3.96	6.41	3.74	0.00	-7.96	5.38	0.45	8.76	4.58
		Oil price	-20%	-2.95	-1.98	-3.21	-1.87	0.00	4.15	-2.72	-0.47	-4.87	-2.81
		Oil price	-50%	-5.89	-3.96	-6.41	-3.73	0.00	8.40	-5.46	-1.10	-10.02	-6.03
		Electricity price	+20%	0.00	0.00	0.00	1.87	1.48	0.08	0.07	0.15	-0.57	-0.51
		Electricity price	-20%	0.00	0.00	0.00	-1.87	-1.48	-0.10	-0.08	-0.19	1.01	0.47
		EV Maintenance cost	+20%	0.00	0.00	0.00	1.26	1.13	0.04	0.03	0.08	-0.47	-0.17
		EV Maintenance cost	-20%	0.00	0.00	0.00	-1.09	-0.93	-0.04	-0.03	-0.08	0.47	0.17
		ICEV Maintenance cost	+20%	3.06	3.24	4.13	0.00	0.00	-0.36	-0.07	-0.45	2.39	1.29
		ICEV Maintenance cost	-20%	-2.51	-2.65	-3.38	0.00	0.00	0.37	0.06	0.47	-2.46	-1.37
		Annual distance	+20%	4.34	3.45	5.08	2.64	1.98	-1.86	0.35	-3.24	1.85	1.27
		Annual distance	-20%	-4.34	-3.45	-5.08	-2.64	-1.98	2.11	0.45	-0.92	-3.78	-3.60
		All ownership costs	+20%	9.41	8.81	11.13	5.45	4.49	-6.16	2.70	-1.29	8.38	5.06
		All ownership costs	-20%	-8.85	-8.22	-10.38	-5.30	-4.31	6.54	-2.95	0.81	-9.04	-5.60

Notes: ICEV denotes Internal Combustion Engine Vehicle, PHEV denotes Plug-in Hybrid Electric Vehicle, and EV denotes electric vehicle.

Suppl. Table 9 | Sensitivity analyses in the current trajectory scenario for the US.

Sensitivity parameters			Var	% Change in Total Cost of Ownership (2035)					%Change in technology shares in 2035				
				Petrol	Diesel	Hybrid	PHEV	EV	Petrol	Diesel	Hybrid	PHEV	EV
Current trajectory scenario	US	**Material cost**											
		Lithium cost	-50%	0.00	0.00	0.00	-0.07	-0.43	-0.05	0.00	-0.07	0.16	0.96
		Lithium cost	+50%	0.00	0.00	0.00	0.01	0.04	0.00	0.00	0.01	-0.01	-0.10
		Lithium cost	+100%	0.00	0.00	0.00	0.07	0.37	0.04	0.00	0.05	-0.13	-0.86
		Cobalt cost	-50%	0.00	0.00	0.00	-0.12	-0.65	-0.06	0.00	-0.08	0.22	1.52
		Cobalt cost	+50%	0.00	0.00	0.00	0.03	0.17	0.02	0.00	0.02	-0.05	-0.38
		Cobalt cost	+100%	0.00	0.00	0.00	0.09	0.51	0.05	0.00	0.07	-0.17	-1.21
		Nickel cost	-50%	0.00	0.00	0.00	-0.14	-0.68	-0.07	0.00	-0.10	0.24	1.60
		Nickel cost	+50%	0.00	0.00	0.00	0.03	0.18	0.02	0.00	0.02	-0.06	-0.40
		Nickel cost	+100%	0.00	0.00	0.00	0.10	0.54	0.06	0.00	0.08	-0.19	-1.26
		Combined cost	-50%	0.00	0.00	0.00	-0.24	-1.66	-0.17	0.00	-0.23	0.57	3.82
		Combined cost	+50%	0.00	0.00	0.00	0.12	0.59	0.07	0.00	0.10	-0.22	-1.30
		Combined cost	+100%	0.00	0.00	0.00	0.23	1.60	0.17	0.00	0.23	-0.56	-3.74
		**Battery improvement**											
		Learning rates	-10%	0.00	0.00	0.00	0.81	4.24	0.33	0.00	0.42	-0.66	-5.80
		Learning rates	+10%	0.00	0.00	0.00	-0.53	-2.78	-0.24	0.00	-0.31	0.78	5.04
		Battery energy density	+10%	0.00	0.00	0.00	-0.27	-1.41	-0.10	0.00	-0.12	0.31	1.98
		Battery energy density	-10%	0.00	0.00	0.00	0.62	3.27	0.09	0.00	0.12	-0.32	-1.92
		Driving range	+50%	0.00	0.00	0.00	0.73	3.83	0.20	0.00	0.26	-0.64	-4.22
		Driving range	-50%	0.00	0.00	0.00	-0.45	-2.36	-0.15	0.00	-0.19	0.48	3.11
		**EV price**											
		Perceived costs	+20%	0.00	0.00	0.00	12.08	11.36	1.35	0.00	1.85	-11.76	-14.82
		Perceived costs	-20%	0.00	0.00	0.00	-10.86	-10.22	-1.25	0.00	-1.79	10.92	12.25
		Markup factor	+10%	0.00	0.00	0.00	8.41	9.05	0.52	0.00	0.69	-4.95	-7.38
		Markup factor	+30%	0.00	0.00	0.00	16.83	18.44	1.02	0.00	1.34	-8.66	-14.58
		EV price	+20%	0.00	0.00	0.00	0.00	20.07	1.51	0.00	2.15	2.31	-22.20
		EV price	-20%	0.00	0.00	0.00	0.00	-21.07	-1.35	0.00	-1.70	-2.58	25.69
		**Ownership cost**											
		Discount rate	+10%	-7.30	-4.86	-5.61	-6.49	-1.05	0.23	0.00	-1.74	-3.67	-1.80
		Discount rate	-10%	8.70	8.21	8.70	9.01	4.56	-0.73	0.00	5.17	5.07	4.56
		Oil price	+20%	3.17	2.16	1.93	2.05	0.00	-0.40	0.00	3.54	4.05	3.33
		Oil price	+50%	6.33	4.32	3.86	4.11	0.00	-0.79	0.00	7.11	7.96	6.57
		Oil price	-20%	-3.17	-2.16	-1.93	-2.05	0.00	0.40	0.00	-3.49	-4.16	-3.42
		Oil price	-50%	-6.33	-4.32	-3.86	-4.10	0.00	0.81	0.00	-6.94	-8.43	-6.92
		Electricity price	+20%	0.00	0.00	0.00	2.06	1.43	0.05	0.00	0.07	-0.51	-0.93
		Electricity price	-20%	0.00	0.00	0.00	-2.06	-1.43	-0.06	0.00	-0.08	0.90	0.92
		EV Maintenance cost	+20%	0.00	0.00	0.00	2.67	2.12	0.03	0.00	0.04	-0.59	-0.47
		EV Maintenance cost	-20%	0.00	0.00	0.00	-2.67	-2.12	-0.03	0.00	-0.04	0.58	0.47
		ICEV Maintenance cost	+20%	4.44	4.71	4.53	0.00	0.00	-0.18	0.00	0.31	2.68	2.21
		ICEV Maintenance cost	-20%	-3.63	-3.85	-3.70	0.00	0.00	0.18	0.00	-0.30	-2.73	-2.25
		Annual distance	+20%	5.19	4.30	3.99	3.21	2.11	-0.69	0.00	6.79	5.28	5.83
		Annual distance	-20%	-5.19	-4.30	-3.99	-3.21	-2.11	0.55	0.00	8.05	-1.69	-1.35
		All ownership costs	+20%	11.45	10.94	9.66	6.70	5.02	-1.27	0.00	8.95	9.59	7.44
		All ownership costs	-20%	-7.00	-6.23	-8.83	-3.77	-2.63	-0.15	0.00	9.23	-9.25	-4.09

Notes: ICEV denotes Internal Combustion Engine Vehicle, PHEV denotes Plug-in Hybrid Electric Vehicle, and EV denotes electric vehicle.

Suppl. Table 10 | Sensitivity analyses in the current trajectory scenario for China.

Sensitivity parameters			Var	% Change in Total Cost of Ownership (2035)					%Change in technology shares in 2035				
				Petrol	Diesel	Hybrid	PHEV	EV	Petrol	Diesel	Hybrid	PHEV	EV
Current trajectory scenario	China	**Material cost**											
		Lithium cost	-50%	0.00	0.00	0.00	-0.11	-0.59	-0.21	-0.12	-0.45	0.10	0.79
		Lithium cost	+50%	0.00	0.00	0.00	0.00	0.06	0.02	0.01	0.04	-0.01	-0.07
		Lithium cost	+100%	0.00	0.00	0.00	0.10	0.49	0.22	0.12	0.49	-0.06	-0.90
		Cobalt cost	-50%	0.00	0.00	0.00	-0.15	-0.87	-0.27	-0.17	-0.63	0.14	1.12
		Cobalt cost	+50%	0.00	0.00	0.00	0.04	0.22	0.07	0.03	0.15	-0.03	-0.30
		Cobalt cost	+100%	0.00	0.00	0.00	0.12	0.69	0.21	0.12	0.51	-0.06	-0.90
		Nickel cost	-50%	0.00	0.00	0.00	-0.18	-0.89	-0.30	-0.18	-0.65	0.15	1.19
		Nickel cost	+50%	0.00	0.00	0.00	0.05	0.23	0.08	0.04	0.17	-0.03	-0.32
		Nickel cost	+100%	0.00	0.00	0.00	0.14	0.71	0.24	0.14	0.52	-0.07	-0.94
		Combined cost	-50%	0.00	0.00	0.00	0.43	-2.16	-0.72	-0.42	-1.57	0.29	2.86
		Combined cost	+50%	0.00	0.00	0.00	0.13	0.52	0.23	0.15	0.54	-0.07	-0.97
		Combined cost	+100%	0.00	0.00	0.00	0.41	2.10	0.71	0.42	1.55	-0.28	-2.82
		**Battery improvement**											
		Learning rates	-10%	0.00	0.00	0.00	0.81	5.58	1.29	0.76	2.96	0.62	-5.03
		Learning rates	+10%	0.00	0.00	0.00	-0.53	-3.65	-0.95	-0.56	-2.15	-0.51	3.69
		Battery energy density	+10%	0.00	0.00	0.00	-0.35	-1.86	-0.38	-0.22	-0.89	-0.24	1.46
		Battery energy density	-10%	0.00	0.00	0.00	0.81	4.33	0.75	0.43	1.79	0.46	-2.88
		Driving range	+50%	0.00	0.00	0.00	0.97	5.04	0.81	0.47	1.95	0.48	-3.19
		Driving range	-50%	0.00	0.00	0.00	-0.59	-3.11	-0.59	-0.34	-1.39	-0.36	2.30
		**EV price**											
		Perceived costs	+20%	0.00	0.00	0.00	13.51	11.07	1.07	0.00	2.38	-8.55	-10.35
		Perceived costs	-20%	0.00	0.00	0.00	-12.33	-11.20	-1.62	0.00	-11.82	-9.38	6.52
		Markup factor	+10%	0.00	0.00	0.00	8.59	9.05	1.35	0.78	3.22	-5.76	-3.98
		Markup factor	+20%	0.00	0.00	0.00	17.18	18.13	2.70	1.56	6.38	-10.27	-8.00
		EV price	+20%	0.00	0.00	0.00	0.00	21.33	5.72	3.38	12.16	8.52	-18.57
		EV price	-20%	0.00	0.00	0.00	0.00	-21.20	-4.72	-2.52	-9.55	-9.78	19.36
		**Ownership cost**											
		Discount rate	+10%	-6.81	-6.06	-5.66	-5.66	-0.51	1.61	5.97	-5.13	-5.57	-4.23
		Discount rate	-10%	8.90	8.70	8.90	8.23	4.34	-3.30	-8.91	5.72	11.24	8.94
		Oil price	+20%	2.71	2.13	2.29	1.63	0.00	-0.84	2.08	0.74	2.84	2.26
		Oil price	+50%	5.43	4.26	4.57	3.27	0.00	-1.63	4.18	1.36	5.54	4.38
		Oil price	-20%	-2.71	-2.13	-2.29	-1.63	0.00	0.89	-2.06	-0.86	-2.97	-2.41
		Oil price	-50%	-5.43	-4.26	-4.57	-3.26	0.00	1.83	-4.09	-1.86	-6.08	-4.97
		Electricity price	+20%	0.00	0.00	0.00	1.65	1.22	0.22	0.13	0.48	-0.07	-0.82
		Electricity price	-20%	0.00	0.00	0.00	-1.65	-1.22	-0.23	-0.14	-0.50	0.37	0.79
		EV Maintenance cost	+20%	0.00	0.00	0.00	2.30	2.18	0.18	0.11	0.40	-0.32	-0.62
		EV Maintenance cost	-20%	0.00	0.00	0.00	-2.30	-2.18	-0.18	-0.11	-0.40	0.31	0.62
		ICEV Maintenance cost	+20%	4.68	4.84	5.23	0.00	0.00	-0.57	0.63	0.03	2.21	1.77
		ICEV Maintenance cost	-20%	-3.83	-3.96	-4.28	0.00	00	0.60	-0.64	-0.08	-2.28	-1.85
		Annual distance	+20%	4.84	4.33	4.66	2.88	2.17	-1.12	-1.91	-0.69	2.62	2.97
		Annual distance	-20%	-4.84	-4.33	-4.66	-2.88	-2.17	0.41	1.95	-2.82	-1.27	-1.03
		All ownership costs	+20%	11.07	10.77	11.09	6.20	5.53	-2.39	-0.95	3.57	8.14	6.20
		All ownership costs	-20%	-6.72	-6.28	-6.73	-3.45	-2.89	1.19	-2.84	-2.02	-4.87	-3.15

Notes: ICEV denotes Internal Combustion Engine Vehicle, PHEV denotes Plug-in Hybrid Electric Vehicle, and EV denotes electric vehicle.

Suppl. Table 11 | Sensitivity analyses in the current trajectory scenario for India

Sensitivity parameters			Var	% Change in Total Cost of Ownership (2035)					%Change in technology shares in 2035				
				Petrol	Diesel	Hybrid	PHEV	EV	Petrol	Diesel	Hybrid	PHEV	EV
Current trajectory scenario	India	**Material cost**											
		Lithium cost	-50%	0.00	0.00	0.00	0.00	0.00	0.00	0.00	0.00	0.00	0.02
		Lithium cost	+50%	0.00	0.00	0.00	0.00	0.03	0.00	0.00	0.01	-0.01	-0.06
		Lithium cost	+100%	0.00	0.00	0.00	0.00	0.42	0.00	0.00	0.00	0.00	-0.14
		Cobalt cost	-50%	0.00	0.00	0.00	0.00	-0.64	0.00	0.00	-0.01	0.00	0.29
		Cobalt cost	+50%	0.00	0.00	0.00	0.00	0.16	0.00	0.00	0.00	0.00	-0.09
		Cobalt cost	+100%	0.00	0.00	0.00	0.00	0.50	0.00	0.00	0.00	0.00	-0.16
		Nickel cost	-50%	0.00	0.00	0.00	0.00	-0.69	0.00	0.00	-0.01	0.00	0.32
		Nickel cost	+50%	0.00	0.00	0.00	0.00	0.17	0.00	0.00	0.00	0.00	-0.10
		Nickel cost	+100%	0.00	0.00	0.00	0.00	0.54	0.00	0.00	0.00	0.00	-0.17
		Combined cost	-50%	0.00	0.00	0.00	0.33	-1.65	0.00	0.00	0.00	0.00	0.18
		Combined cost	+50%	0.00	0.00	0.00	0.00	0.46	0.00	0.00	0.00	0.00	-0.16
		Combined cost	+100%	0.00	0.00	0.00	0.31	1.60	0.00	0.00	0.00	0.00	-0.17
		**Battery improvement**											
		Learning rates	-10%	0.00	0.00	0.00	0.89	4.23	0.02	0.01	0.04	0.00	-3.03
		Learning rates	+10%	0.00	0.00	0.00	-0.59	-2.77	-0.01	-0.01	-0.02	0.00	2.25
		Battery energy density	+10%	0.00	0.00	0.00	-0.29	-1.41	-0.01	0.00	-0.01	0.00	0.89
		Battery energy density	-10%	0.00	0.00	0.00	0.68	3.27	0.01	0.01	0.03	0.00	-1.72
		Driving range	+50%	0.00	0.00	0.00	0.81	3.82	0.01	0.01	0.03	0.00	-1.89
		Driving range	-50%	0.00	0.00	0.00	-0.50	-2.36	-0.01	-0.01	-0.02	0.00	1.38
		**EV price**											
		Perceived costs	+20%	0.00	0.00	0.00	0.07	2.31	0.09	0.05	0.16	0.00	-4.87
		Perceived costs	-20%	0.00	0.00	0.00	-0.02	-4.39	1.19	0.00	-0.36	0.00	6.52
		Markup factor	+10%	0.00	0.00	0.00	8.52	9.19	0.04	0.03	0.100	0.00	-6.50
		Markup factor	+20%	0.00	0.00	0.00	17.04	18.41	0.08	0.05	0.19	-0.0	-13.90
		EV price	+20%	0.00	0.00	0.00	0.00	21.12	0.07	0.05	0.16	0.00	-14.52
		EV price	-20%	0.00	0.00	0.00	0.00	-21.34	-0.08	-0.04	-0.12	0.00	11.82
		**Ownership cost**											
		Discount rate	+10%	-10.46	-10.02	-6.02	-6.02	-0.62	2.07	7.80	-0.61	0.00	-11.00
		Discount rate	-10%	7.42	7.12	7.42	8.58	4.75	-3.21	-5.03	5.75	0.00	10.95
		Oil price	+20%	4.39	3.99	2.60	0.00	0.00	-0.83	4.20	4.48	0.00	4.29
		Oil price	+50%	8.78	7.99	5.21	0.00	0.00	-1.62	8.41	9.62	0.00	9.24
		Oil price	-20%	-4.39	-3.99	-2.60	0.00	0.00	1.78	-8.38	-10.14	0.00	-4.99
		Oil price	-50%	-8.78	-7.99	-5.21	0.00	0.00	1.78	-8.38	-9.24	0.00	-8.22
		Electricity price	+20%	0.00	0.00	0.00	0.71	0.95	0.02	0.01	0.04	0.00	-2.79
		Electricity price	-20%	0.00	0.00	0.00	-0.71	-0.95	-0.02	-0.01	-0.04	0.00	2.71
		EV Maintenance cost	+20%	0.00	0.00	0.00	1.90	1.82	0.01	0.01	0.03	0.00	-2.42
		EV Maintenance cost	-20%	0.00	0.00	0.00	-1.90	-1.82	-0.01	-0.01	-0.03	0.00	2.42
		ICEV Maintenance cost	+20%	6.70	6.87	4.52	0.00	0.00	0.18	-2.28	3.74	0.00	6.61
		ICEV Maintenance cost	-20%	-5.48	-5.62	-3.70	0.00	0.00	-0.16	2.34	-3.11	0.00	-6.57
		Annual distance	+20%	7.44	7.12	4.15	3.01	1.82	-0.43	-4.85	3.25	0.00	1.95
		Annual distance	-20%	-7.44	-7.12	-4.15	-3.01	-1.82	0.12	1.84	-2.14	0.00	-4.16
		All ownership costs	+20%	15.77	15.66	10.31	7.06	4.68	-0.07	-4.28	11.49	0.00	11.23
		All ownership costs	-20%	-10.22	-9.97	-6.44	-4.02	-2.47	0.63	-1.25	-10.68	0.00	-10.86

Notes: ICEV denotes Internal Combustion Engine Vehicle, PHEV denotes Plug-in Hybrid Electric Vehicle, and EV denotes electric vehicle.

Suppl. Table 12 | Sensitivity analyses in the policy combinations scenario for Europe

Sensitivity parameters			Var	% Change in Total Cost of Ownership (2035)					%Change in technology shares in 2035				
				Petrol	Diesel	Hybrid	PHEV	EV	Petrol	Diesel	Hybrid	PHEV	EV
Current Trajectories +Taxes +Subsidies + Regulations + EV mandate	Europe	**Material cost**											
		Lithium cost	-50%	0.00	0.00	0.00	-0.00	-0.06	0.00	0.00	0.00	0.00	0.02
		Lithium cost	+50%	0.00	0.00	0.00	0.01	0.08	0.00	0.00	0.00	-0.01	-0.01
		Lithium cost	+100 %	0.00	0.00	0.00	0.03	0.18	0.00	0.00	0.00	-0.04	-0.03
		Cobalt cost	-50%	0.00	0.00	0.00	-0.04	-0.30	-0.00	-0.00	-0.00	0.07	0.02
		Cobalt cost	+50%	0.00	0.00	0.00	0.02	0.11	0.00	0.00	0.00	0.01	-0.02
		Cobalt cost	+100 %	0.00	0.00	0.00	0.04	0.22	0.00	0.00	0.00	0.06	-0.02
		Nickel cost	-50%	0.00	0.00	0.00	-0.06	-0.34	-0.00	-0.00	-0.00	0.10	0.03
		Nickel cost	+50%	0.00	0.00	0.00	0.02	0.12	0.00	0.00	0.00	0.03	-0.02
		Nickel cost	+100 %	0.00	0.00	0.00	0.05	0.27	0.00	0.00	0.00	0.09	-0.02
		Combined cost	-50%	0.00	0.00	0.00	-0.08	-0.45	-0.00	-0.00	-0.00	0.15	0.05
		Combined cost	+50%	0.00	0.00	0.00	0.03	0.21	0.00	0.00	0.00	0.05	-0.03
		Combined cost	+100 %	0.00	0.00	0.00	0.15	0.79	0.00	0.00	0.00	0.15	-0.09
		**Battery improvement**											
		Learning rates	-10%	0.00	0.00	0.00	-1.55	2.92	0.00	0.00	0.00	0.76	-0.20
		Learning rates	+10%	0.00	0.00	0.00	-0.29	0.55	0.00	0.00	0.00	-0.43	0.11
		Battery energy density	+10%	0.00	0.00	0.00	-0.12	-0.66	0.00	0.00	0.00	-0.16	0.04
		Battery energy density	-10%	0.00	0.00	0.00	0.27	1.46	0.00	0.00	0.00	0.32	-0.08
		Driving range	+50%	0.00	0.00	0.00	0.32	1.71	0.00	0.00	0.00	0.36	-0.09
		Driving range	-50%	0.00	0.00	0.00	-0.20	-1.07	0.00	0.00	0.00	-0.25	0.08
		**EV price**											
		Perceived costs	+20%	0.00	0.00	0.00	10.45	14.97	0.00	0.00	0.00	1.60	-0.43
		Perceived costs	-20%	0.00	0.00	0.00	-9.12	-14.25	-0.13	0.00	-0.90	0.85	0.22
		Markup factor	+10%	0.00	0.00	0.00	8.33	9.01	0.00	0.00	0.00	-0.31	-0.07
		Markup factor	+20%	0.00	0.00	0.00	17.43	18.00	0.00	0.00	0.00	-0.60	0.15
		EV price	+20%	0.00	0.00	0.00	0.00	19.98	0.00	0.00	0.00	4.56	-1.09
		EV price	-20%	0.00	0.00	0.00	0.00	-20.34	0.00	0.00	0.00	-5.20	1.43
		**Ownership cost**											
		Discount rate	+10%	-6.10	-4.86	-7.15	-5.49	-1.54	0.22	0.08	3.80	-0.11	-0.05
		Discount rate	-10%	8.21	8.21	8.21	8.21	6.54	-0.24	-0.09	-4.79	0.27	0.08
		Oil price	+20%	2.95	1.98	3.21	1.88	0.00	-0.04	-0.01	0.45	0.00	0.00
		Oil price	+50%	5.89	3.96	6.41	3.75	0.00	-0.07	-0.03	0.92	0.00	0.00
		Oil price	-20%	-2.95	-1.98	-3.21	-1.88	0.00	0.04	0.01	0.41	0.00	0.00
		Oil price	-50%	-5.89	-3.96	-6.41	-3.75	0.00	0.07	0.03	-0.84	0.00	0.00
		Electricity price	+20%	0.00	0.00	0.00	1.64	1.49	0.00	0.00	0.00	0.09	-0.02
		Electricity price	-20%	0.00	0.00	0.00	-1.64	-1.49	0.00	0.00	0.00	-0.08	0.02
		EV Maintenance cost	+20%	0.00	0.00	0.00	1.26	1.14	0.00	0.00	0.00	-0.03	0.01
		EV Maintenance cost	-20%	0.00	0.00	0.00	-1.10	-0.93	0.00	0.00	0.00	0.03	-0.01
		ICEV Maintenance cost	+20%	3.06	3.24	4.13	0.00	0.00	0.00	0.00	0.01	0.00	0.00
		ICEV Maintenance cost	-20%	-2.51	-2.65	-3.38	0.00	0.00	0.00	0.00	-0.01	0.00	0.00
		Annual distance	+20%	4.34	3.45	5.08	2.65	1.98	-0.03	-0.83	-0.27	1.33	0.23
		Annual distance	-20%	-4.34	-3.45	-5.08	-2.65	-1.98	0.03	0.86	-0.35	-0.76	-0.38
		All ownership costs	+20%	9.41	8.81	11.13	5.48	4.63	-0.07	-0.02	0.50	-0.22	0.06
		All ownership costs	-20%	-8.85	-8.22	-10.38	-5.32	-4.42	0.07	0.03	-0.47	0.33	-0.09

Notes: ICEV denotes Internal Combustion Engine Vehicle, PHEV denotes Plug-in Hybrid Electric Vehicle, and EV denotes electric vehicle.

Suppl. Table 13 | Sensitivity analyses in the policy combinations scenario for the US.

Scenario	Region	Sensitivity parameters	Var	% Change in Total Cost of Ownership (2035)					%Change in technology shares in 2035				
				Petrol	Diesel	Hybrid	PHEV	EV	Petrol	Diesel	Hybrid	PHEV	EV
Current Trajectories +Taxes +Subsidies + Regulations + EV mandate	US	**Material cost**											
		Lithium cost	-50%	0.00	0.00	0.00	-0.02	-0.11	-0.00	0.00	-0.03	0.03	0.03
		Lithium cost	+50%	0.00	0.00	0.00	0.02	0.12	0.00	0.00	0.02	-0.05	-0.03
		Lithium cost	+100%	0.00	0.00	0.00	0.06	0.28	0.00	0.00	0.05	-0.08	-0.06
		Cobalt cost	-50%	0.00	0.00	0.00	-0.08	-0.46	-0.00	-0.00	-0.00	0.12	-0.12
		Cobalt cost	+50%	0.00	0.00	0.00	0.02	0.17	0.02	0.00	0.02	-0.05	-0.38
		Cobalt cost	+100%	0.00	0.00	0.00	0.07	0.38	0.00	0.00	0.00	-0.09	-0.03
		Nickel cost	-50%	0.00	0.00	0.00	-0.10	-0.52	-0.00	-0.00	-0.00	0.14	-0.13
		Nickel cost	+50%	0.00	0.00	0.00	0.03	0.18	0.02	0.00	0.02	-0.06	-0.40
		Nickel cost	+100%	0.00	0.00	0.00	0.08	0.41	0.00	0.00	0.00	-0.10	-0.03
		Combined cost	-50%	0.00	0.00	0.00	-0.16	-1.04	-0.00	0.00	-0.00	0.21	-0.25
		Combined cost	+50%	0.00	0.00	0.00	0.12	0.59	0.07	0.00	0.00	0.21	-0.05
		Combined cost	+100%	0.00	0.00	0.00	0.23	1.21	0.00	0.00	0.00	0.33	-0.13
		**Battery improvement**											
		Learning rates	-10%	0.00	0.00	0.00	-2.51	4.38	0.02	0.00	0.04	1.07	-0.33
		Learning rates	+10%	0.00	0.00	0.00	-0.52	0.78	-0.02	0.00	-0.04	-0.60	0.20
		Battery energy density	+10%	0.00	0.00	0.00	-0.19	-1.02	-0.01	0.00	-0.01	-0.22	0.07
		Battery energy density	-10%	0.00	0.00	0.00	0.42	2.26	0.01	0.00	0.02	0.46	-0.14
		Driving range	+50%	0.00	0.00	0.00	0.50	2.64	0.01	0.00	0.04	0.79	-0.19
		Driving range	-50%	0.00	0.00	0.00	-0.31	-1.64	-0.02	0.00	-0.03	-0.87	0.27
		**EV price**											
		Perceived costs	+20%	0.00	0.00	0.00	12.05	0.48	0.00	0.77	-0.76	-0.57	0.48
		Perceived costs	-20%	0.00	0.00	0.00	-10.88	-0.45	0.00	-0.69	0.71	0.53	-0.45
		Markup factor	+10%	0.00	0.00	0.00	8.01	8.76	0.01	0.00	0.02	-0.18	-0.03
		Markup factor	+20%	0.00	0.00	0.00	16.46	17.46	0.03	0.00	0.04	-0.35	-0.05
		EV price	+20%	0.00	0.00	0.00	0.00	20.02	0.30	0.00	0.35	6.05	-5.75
		EV price	-20%	0.00	0.00	0.00	0.00	-21.13	-0.23	0.00	-0.37	-5.14	4.78
		**Ownership cost**											
		Discount rate	+10%	-7.30	-6.06	-5.61	-6.71	-1.05	0.10	0.00	-0.43	-0.90	-0.17
		Discount rate	-10%	8.70	7.75	7.66	8.70	5.56	-0.15	0.00	0.63	1.62	0.32
		Oil price	+20%	3.17	2.16	1.93	2.07	0.00	-0.09	0.00	0.49	0.08	0.04
		Oil price	+50%	6.33	4.32	3.86	4.14	0.00	-0.19	0.00	0.97	0.16	0.08
		Oil price	-20%	-3.17	-2.16	-1.93	-2.07	0.00	0.08	0.00	0.42	0.08	0.04
		Oil price	-50%	-6.33	-4.32	-3.86	-4.14	0.00	0.20	0.00	-0.99	-0.17	-0.09
		Electricity price	+20%	0.00	0.00	0.00	1.70	1.45	0.00	0.00	0.01	0.07	-0.03
		Electricity price	-20%	0.00	0.00	0.00	-1.70	-1.45	0.00	0.00	0.01	-0.06	-0.04
		EV Maintenance cost	+20%	0.00	0.00	0.00	2.68	2.15	0.01	0.00	0.01	-0.01	-0.01
		EV Maintenance cost	-20%	0.00	0.00	0.00	-2.68	-2.15	-0.01	0.00	0.00	0.01	0.01
		ICEV Maintenance cost	+20%	4.44	4.71	4.53	0.00	0.00	-0.04	0.00	0.04	0.06	0.03
		ICEV Maintenance cost	-20%	-3.63	-3.85	-3.70	0.00	0.00	0.03	0.00	-0.04	-0.05	-0.03
		Annual distance	+20%	5.19	4.30	3.99	3.22	2.11	-0.01	0.00	0.18	0.32	0.07
		Annual distance	-20%	-5.19	-4.30	-3.99	-3.22	-2.11	0.03	0.00	1.26	-0.19	-0.08
		All ownership costs	+20%	11.45	10.94	9.66	6.75	5.24	-0.31	0.00	1.70	0.07	0.18
		All ownership costs	-20%	-7.00	-6.23	-8.83	-3.80	-2.77	-0.11	0.00	1.85	-0.16	-0.06

Notes: ICEV denotes Internal Combustion Engine Vehicle, PHEV denotes Plug-in Hybrid Electric Vehicle, and EV denotes electric vehicle.

Suppl. Table 14 | Sensitivity analyses in the policy combinations scenario for China

Sensitivity parameters			Var	% Change in Total Cost of Ownership (2035)					%Change in technology shares in 2035				
				Petrol	Diesel	Hybrid	PHEV	EV	Petrol	Diesel	Hybrid	PHEV	EV
Current Trajectories +Taxes +Subsidies + Regulations + EV mandate	China	**Material cost**											
		Lithium cost	-50%	0.00	0.00	0.00	-0.04	-0.18	-0.00	-0.00	-0.00	0.00	0.02
		Lithium cost	+50%	0.00	0.00	0.00	0.05	0.16	0.00	0.00	0.01	-0.00	-0.03
		Lithium cost	+100%	0.00	0.00	0.00	0.08	0.37	0.01	0.01	0.03	0.01	-0.01
		Cobalt cost	-50%	0.00	0.00	0.00	-0.11	-0.56	-0.02	-0.01	-0.04	-0.02	0.01
		Cobalt cost	+50%	0.00	0.00	0.00	0.04	0.22	0.07	0.03	0.15	-0.03	-0.30
		Cobalt cost	+100%	0.00	0.00	0.00	0.05	0.50	0.01	0.01	0.03	-0.01	-0.02
		Nickel cost	-50%	0.00	0.00	0.00	-0.13	-0.69	-0.03	-0.02	-0.05	-0.02	0.02
		Nickel cost	+50%	0.00	0.00	0.00	0.05	0.23	0.08	0.04	0.17	-0.03	-0.32
		Nickel cost	+100%	0.00	0.00	0.00	0.07	0.54	0.02	0.01	0.04	-0.02	-0.02
		Combined cost	-50%	0.00	0.00	0.00	-0.20	-0.98	-0.06	-0.05	-0.09	0.05	0.05
		Combined cost	+50%	0.00	0.00	0.00	0.10	0.42	0.12	0.09	0.20	-0.07	-0.77
		Combined cost	+100%	0.00	0.00	0.00	0.30	1.59	0.06	0.04	0.12	0.05	-0.06
		**Battery improvement**											
		Learning rates	-10%	0.00	0.00	0.00	0.73	5.83	0.14	0.08	0.26	0.11	-0.12
		Learning rates	+10%	0.00	0.00	0.00	-0.59	-3.30	-0.12	-0.07	-0.23	-0.09	0.11
		Battery energy density	+10%	0.00	0.00	0.00	-0.25	-1.35	0.01	0.00	0.02	0.46	-0.14
		Battery energy density	-10%	0.00	0.00	0.00	0.56	3.00	-0.05	0.00	-0.10	-0.04	0.05
		Driving range	+50%	0.00	0.00	0.00	0.65	3.49	0.08	0.06	0.15	0.07	-0.08
		Driving range	-50%	0.00	0.00	0.00	-0.41	-2.18	-0.10	-0.05	-0.18	-0.08	0.09
		**EV price**											
		Perceived costs	+20%	0.00	0.00	0.00	13.66	6.94	0.31	0.18	0.72	-2.85	-0.68
		Perceived costs	-20%	0.00	0.00	0.00	-12.41	-10.33	0.22	1.07	0.54	2.72	0.64
		Markup factor	+10%	0.00	0.00	0.00	8.23	9.34	0.06	0.03	0.12	-0.05	-0.04
		Markup factor	+20%	0.00	0.00	0.00	17.35	17.89	0.12	0.07	0.23	-0.10	-0.08
		EV price	+20%	0.00	0.00	0.00	0.00	21.90	0.71	0.35	1.62	1.12	-1.57
		EV price	-20%	0.00	0.00	0.00	0.00	-20.02	-0.81	-0.46	-1.52	-1.05	1.09
		**Ownership cost**											
		Discount rate	+10%	-6.81	-6.10	-6.56	-5.97	-0.51	0.48	3.35	2.06	-0.26	-0.24
		Discount rate	-10%	8.90	8.90	8.90	8.90	3.34	-0.65	-6.73	-2.68	0.35	0.31
		Oil price	+20%	2.71	2.13	2.29	1.65	0.00	-0.19	0.71	0.21	0.08	0.11
		Oil price	+50%	5.43	4.26	4.57	3.31	0.00	-0.37	1.43	0.40	0.16	0.22
		Oil price	-20%	-2.71	-2.13	-2.29	-1.65	0.00	0.18	0.68	-0.20	-0.08	-0.10
		Oil price	-50%	-5.43	-4.26	-4.57	-3.31	0.00	0.42	-1.39	-0.47	-0.17	-0.25
		Electricity price	+20%	0.00	0.00	0.00	1.17	1.23	0.04	0.02	0.08	0.03	-0.04
		Electricity price	-20%	0.00	0.00	0.00	-1.17	-1.23	-0.05	-0.03	-0.08	-0.01	0.04
		EV Maintenance cost	+20%	0.00	0.00	0.00	2.31	2.23	0.04	0.02	0.07	0.01	-0.03
		EV Maintenance cost	-20%	0.00	0.00	0.00	-2.31	-2.23	-0.04	-0.02	-0.07	-0.01	0.03
		ICEV Maintenance cost	+20%	4.68	4.84	5.23	0.00	0.00	-0.13	0.27	0.04	0.06	0.09
		ICEV Maintenance cost	-20%	-3.83	-3.96	-4.28	0.00	0.00	0.13	-0.26	-0.04	-0.07	-0.10
		Annual distance	+20%	4.84	4.33	4.66	2.89	2.17	0.15	0.74	-1.97	-0.01	-0.12
		Annual distance	-20%	-4.84	-4.33	-4.66	-2.89	-2.17	0.48	1.89	-2.54	-0.07	-0.31
		All ownership costs	+20%	11.07	10.77	11.09	6.26	5.84	-0.55	-0.16	0.95	0.23	0.52
		All ownership costs	-20%	-6.72	-6.28	-6.73	-3.49	-3.08	0.28	-0.97	-0.47	-0.17	-0.16

Notes: ICEV denotes Internal Combustion Engine Vehicle, PHEV denotes Plug-in Hybrid Electric Vehicle, and EV denotes electric vehicle.

Suppl. Table 15 | Sensitivity analyses in the policy combinations scenario for India

Sensitivity parameters			Var	% Change in Total Cost of Ownership (2035)					%Change in technology shares in 2035				
				Petrol	Diesel	Hybrid	PHEV	EV	Petrol	Diesel	Hybrid	PHEV	EV
Current Trajectories +Taxes +Subsidies + Regulations + EV mandate	India	**Material cost**											
		Lithium cost	-50%	0.00	0.00	0.00	-0.05	-0.07	-0.00	-0.00	-0.00	0.00	0.00
		Lithium cost	+50%	0.00	0.00	0.00	0.04	0.15	0.00	0.00	0.00	-0.00	-0.00
		Lithium cost	+100%	0.00	0.00	0.00	0.06	0.28	0.00	0.00	0.00	0.00	-0.00
		Cobalt cost	-50%	0.00	0.00	0.00	0.00	-0.00	0.00	0.00	0.00	0.00	-0.00
		Cobalt cost	+50%	0.00	0.00	0.00	0.00	0.16	0.00	0.00	0.00	0.00	-0.09
		Cobalt cost	+100%	0.00	0.00	0.00	0.00	-0.00	0.00	0.00	0.00	0.00	-0.00
		Nickel cost	-50%	0.00	0.00	0.00	-0.11	-0.52	0.00	0.00	-0.00	0.00	0.00
		Nickel cost	+50%	0.00	0.00	0.00	0.00	0.17	0.00	0.00	0.00	0.00	-0.10
		Nickel cost	+100%	0.00	0.00	0.00	0.09	0.41	0.00	0.00	0.00	0.00	-0.00
		Combined cost	-50%	0.00	0.00	0.00	-0.12	-1.23	0.00	0.00	0.00	0.00	0.00
		Combined cost	+50%	0.00	0.00	0.00	0.10	0.56	0.00	0.00	0.00	0.00	0.00
		Combined cost	+100%	0.00	0.00	0.00	0.26	1.21	0.00	0.00	0.00	0.00	0.00
		**Battery improvement**											
		Learning rates	-10%	0.00	0.00	0.00	0.93	4.50	0.00	0.00	0.00	0.00	0.00
		Learning rates	+10%	0.00	0.00	0.00	-0.50	-2.39	0.00	0.00	0.00	0.00	0.00
		Battery energy density	+10%	0.00	0.00	0.00	-0.21	-1.01	-0.01	0.00	-0.01	0.00	0.89
		Battery energy density	-10%	0.00	0.00	0.00	0.46	2.25	0.01	0.01	0.03	0.00	-1.72
		Driving range	+50%	0.00	0.00	0.00	0.55	2.63	0.00	0.00	0.00	0.00	0.00
		Driving range	-50%	0.00	0.00	0.00	-0.34	-1.64	0.00	0.00	0.00	0.00	0.00
		**EV price**											
		Perceived costs	+20%	0.00	0.00	0.00	0.00	6.88	0.00	0.00	0.00	0.00	-0.56
		Perceived costs	-20%	0.00	0.00	0.00	0.00	-4.67	0.00	0.00	0.00	0.00	0.74
		Markup factor	+10%	0.00	0.00	0.00	8.62	9.10	0.02	0.01	0.04	0.00	-0.20
		Markup factor	+20%	0.00	0.00	0.00	16.98	18.21	0.04	0.03	0.08	0.00	-0.40
		EV price	+20%	0.00	0.00	0.00	0.00	19.20	0.00	0.00	0.00	0.00	0.63
		EV price	-20%	0.00	0.00	0.00	0.00	-19.88	0.00	0.00	0.00	0.00	0.72
		**Ownership cost**											
		Discount rate	+10%	-10.46	-10.02	-6.56	-6.27	-2.65	1.10	1.12	0.29	0.00	-0.10
		Discount rate	-10%	7.42	7.42	7.42	7.42	4.92	-0.15	-1.45	-0.51	0.00	0.05
		Oil price	+20%	4.39	3.99	2.60	0.71	0.00	-0.20	0.76	5.17	0.00	0.00
		Oil price	+50%	8.78	7.99	5.21	1.42	0.00	-0.41	1.54	10.36	0.00	0.00
		Oil price	-20%	-4.39	-3.99	-2.60	-0.71	0.00	0.25	0.70	5.56	0.00	0.00
		Oil price	-50%	-8.78	-7.99	-5.21	-1.42	0.00	0.39	-1.48	-10.64	0.00	0.00
		Electricity price	+20%	0.00	0.00	0.00	0.31	1.05	0.00	0.00	0.00	0.00	0.00
		Electricity price	-20%	0.00	0.00	0.00	-0.31	-1.05	0.00	0.00	0.00	0.00	0.00
		EV Maintenance cost	+20%	0.00	0.00	0.00	1.90	1.85	0.04	0.02	0.07	0.01	-0.03
		EV Maintenance cost	-20%	0.00	0.00	0.00	-1.90	-1.85	-0.04	-0.02	-0.07	-0.01	0.03
		ICEV Maintenance cost	+20%	6.70	6.87	4.52	0.00	0.00	-0.04	-0.03	2.90	0.00	0.00
		ICEV Maintenance cost	-20%	-5.48	-5.62	-3.70	0.00	0.00	0.03	0.00	-0.04	-0.05	-0.03
		Annual distance	+20%	7.44	7.12	4.15	3.02	1.82	-0.31	2.90	0.75	0.00	-0.11
		Annual distance	-20%	-7.44	-7.12	-4.15	-3.02	-1.82	0.31	1.24	-0.55	0.00	0.08
		All ownership costs	+20%	15.77	15.66	10.31	7.11	4.89	-0.30	0.57	0.85	0.00	0.00
		All ownership costs	-20%	-10.22	-9.97	-6.44	-4.05	-2.61	0.25	-0.71	-1.12	0.00	0.00

Notes: ICEV denotes Internal Combustion Engine Vehicle, PHEV denotes Plug-in Hybrid Electric Vehicle, and EV denotes electric vehicle.

Suppl Table 16 | Sensitivity analyses on the stringency of policy instruments, China

	Sensitivity parameters	Change in fleet shares in 2050%				
		Petrol	Diesel	HEV	EV	PHEV
1	VT/RT+10%	-1.2%	0.0%	0.0%	7.1%	-3.4%
2	VT/RT-10%	1.4%	0.0%	0.0%	-7.4%	3.7%
3	EV_sub+10%	NA	NA	NA	NA	NA
4	EV_sub-10%	NA	NA	NA	NA	NA
5	VT/RT+10%&Regulation 2 years later	6.8%	1.5%	2.7%	-23.4%	3.2%
6	VT/RT-10%&Regulation 2 years later	3.0%	1.4%	2.5%	-29.4%	5.6%
7	VT/RT+10%&Regulation 2 years earlier	-8.4%	-1.5%	-2.8%	18.8%	4.4%
8	VT/RT-10%&Regulation 2 years earlier	-9.4%	-1.5%	-2.9%	17.8%	5.1%
9	5 & EV mandate+10%	-5.8%	1.3%	2.3%	-19.9%	2.7%
10	6 & EV mandate+10%	-2.5%	1.2%	2.2%	-25.0%	4.8%
11	7 & EV mandate+10%	-8.4%	-0.5%	-1.4%	23.2%	-9.4%
12	8 & EV mandate+10%	-8.1%	-0.6%	-1.5%	22.9%	-9.3%
13	5 & EV mandate-10%	5.8%	1.3%	2.3%	-19.8%	2.7%
14	6 & EV mandate-10%	2.5%	1.2%	2.1%	-24.8%	4.7%
15	7 & EV mandate -10%	9.0%	-1.4%	-3.5%	-8.2%	11.6%
16	8 & EV mandate-10%	8.5%	-1.4%	-3.5%	-8.6%	11.6%

Notes: Notes: PHEV denotes Plug-in Hybrid Electric Vehicle, HEV hybrid vehicles, EV denotes electric vehicle., VT = vehicle tax, RT = road tax

Suppl Table 17 | Sensitivity analyses on the stringency of policy instruments, US

	Sensitivity parameters	Change in shares in 2050%				
		Petrol	Diesel	HEV	EV	PHEV
1	VT/RT+10%	-0.09%	0.00%	0.00%	0.29%	-0.20%
2	VT/RT-10%	0.11%	0.00%	0.00%	-0.37%	-0.21%
3	EV_sub+10%	0.14%	0.00%	0.00%	0.43%	-0.29%
4	EV_sub-10%	-0.08%	0.00%	0.00%	-0.23%	0.15%
5	VT/RT+10% & Regulation 2 years later	0.01%	0.00%	0.00%	0.01%	0.15%
6	VT/RT-10% & Regulation 2 years later	0.00%	0.00%	0.00%	0.00%	0.48%
7	EV_sub+10% & Regulation 2 years later	0.02%	0.00%	0.00%	0.19%	0.14%
8	EV_sub-10% & Regulation 2 years later	-0.01%	0.00%	0.00%	-0.07%	-0.10%
9	VT/RT+10% & Regulation 2 years earlier	0.01%	0.00%	0.00%	0.05%	0.18%
10	VT/RT-10% & Regulation 2 years earlier	0.01%	0.00%	0.00%	0.05%	0.18%
11	EV_sub+10% & Regulation 2 years earlier	0.02%	0.00%	0.00%	0.03%	0.18%
12	EV_sub-10% & Regulation 2 years earlier	-0.01%	0.00%	0.00%	-0.01%	-0.07%
13	9 & EV mandate+10%	-0.54%	0.01%	0.10%	2.11%	0.07%
14	10 & EV mandate+10%	-0.54%	0.01%	0.10%	2.02%	0.07%
15	11 & EV mandate+10%	-0.55%	0.01%	0.10%	2.11%	0.07%
16	12 & stringency+10%	-0.54%	0.01%	0.10%	2.04%	0.07%
17	9 & EV mandate-10%	0.55%	-0.01%	-0.10%	-2.39%	-0.07%
18	10 & EV mandate-10%	0.56%	-0.01%	-0.10%	-2.49%	-0.07%
19	11 & EV mandate-10%	0.55%	-0.01%	-0.10%	-2.39%	-0.07%
20	12 & EV mandate-10%	0.56%	-0.02%	-0.10%	-2.46%	-0.07%

Notes: Notes: PHEV denotes Plug-in Hybrid Electric Vehicle, HEV hybrid vehicles, EV denotes electric vehicle., VT = vehicle tax, RT = road tax

Suppl Table 18 | Sensitivity analyses on the stringency of policy instruments, EU

	Sensitivity parameters	Change in shares in 2050%				
		Petrol	Diesel	HEV	EV	PHEV
1	VT/RT+10%	-0.98%	0.00%	0.02%	3.03%	-2.09%
2	VT/RT-10%	1.19%	0.00%	0.02%	-4.12%	-2.17%
3	EV_sub+10%	1.51%	0.01%	0.02%	4.70%	-3.09%
4	EV_sub-10%	-0.79%	0.00%	-0.01%	-2.48%	1.63%
5	VT/RT+10% & Regulation 2 years later	0.08%	0.00%	0.00%	1.23%	1.41%
6	VT/RT-10% & Regulation 2 years later	-0.04%	0.00%	-0.01%	-0.57%	4.56%
7	EV_sub+10% & Regulation 2 years later	0.25%	0.01%	0.02%	16.03%	1.27%
8	EV_sub-10% & Regulation 2 years later	-0.09%	0.00%	-0.01%	-8.39%	-0.98%
9	VT/RT+10% & Regulation 2 years earlier	0.08%	0.00%	0.01%	5.94%	1.87%
10	VT/RT-10% & Regulation 2 years earlier	0.08%	0.00%	0.01%	5.94%	1.87%
11	EV_sub+10% & Regulation 2 years earlier	0.26%	0.01%	0.03%	4.02%	1.88%
12	EV_sub-10% & Regulation 2 years earlier	-0.09%	0.00%	-0.01%	-1.38%	-0.88%
13	9 & EV mandate+10%	-4.59%	0.12%	1.17%	25.50%	0.82%
14	10 & EV mandate+10%	-4.56%	0.12%	1.17%	24.35%	0.82%
15	11 & EV mandate+10%	-4.65%	0.12%	0.92%	25.52%	0.83%
16	12 & EV mandate+10%	-4.34%	0.12%	0.91%	24.67%	0.81%
17	9 & EV mandate-10%	4.49%	-0.12%	-0.83%	-22.48%	-0.66%
18	10 & EV mandate-10%	4.52%	-0.12%	-0.83%	-21.06%	-0.59%
19	11 & EV mandate-10%	4.44%	-0.12%	-0.82%	-20.27%	-0.58%
20	12 & EV mandate-10%	4.74%	-0.13%	-0.85%	-20.82%	-0.60%

Notes: Notes: PHEV denotes Plug-in Hybrid Electric Vehicle, HEV hybrid vehicles, EV denotes electric vehicle., VT = vehicle tax, RT = road tax

Suppl Table 19 | Sensitivity analyses on the stringency of policy instruments, India

	Sensitivity parameters	Change in shares in 2050%				
		Petrol	Diesel	HEV	EV	PHEV
1	VT/RT+10%	-0.01%	0.00%	0.00%	0.02%	-0.02%
2	VT/RT-10%	0.01%	0.00%	0.00%	-0.02%	0.02%
3	EV_sub+10%	-0.09%	0.00%	0.00%	0.29%	-0.20%
4	EV_sub-10%	0.03%	0.00%	0.00%	-0.11%	0.07%
5	VT/RT+10% & Regulation 2 years later	0.00%	0.00%	0.00%	0.00%	0.07%
6	VT/RT-10% & Regulation 2 years later	0.00%	0.00%	0.00%	0.00%	0.06%
7	EV_sub+10% & Regulation 2 years later	0.00%	0.00%	0.00%	0.00%	0.10%
8	EV_sub-10% & Regulation 2 years later	0.00%	0.00%	0.00%	0.00%	0.02%
9	VT/RT+10% & Regulation 2 years earlier	0.00%	0.00%	0.00%	0.00%	-0.75%
10	VT/RT-10% & Regulation 2 years earlier	0.00%	0.00%	0.00%	0.00%	-0.75%
11	EV_sub+10% & Regulation 2 years earlier	0.00%	0.00%	0.00%	0.00%	-0.72%
12	EV_sub-10% & Regulation 2 years earlier	0.00%	0.00%	0.00%	0.00%	-0.08%
13	9 & EV mandate+10%	-0.06%	0.00%	0.01%	0.08%	0.10%
14	10 & EV mandate+10%	-0.06%	0.00%	0.01%	0.08%	0.10%
15	11 & EV mandate+10%	-0.07%	0.00%	0.01%	0.08%	0.10%
16	12 & EV mandate+10%	-0.06%	0.00%	0.01%	0.08%	0.10%
17	9 & EV mandate-10%	0.07%	0.00%	-0.01%	-0.08%	0.74%
18	10 & EV mandate-10%	0.07%	0.00%	-0.01%	-0.08%	0.74%
19	11 & EV mandate-10%	0.07%	0.00%	-0.01%	-0.08%	0.76%
20	12 & EV mandate-10%	0.07%	0.00%	-0.01%	-0.08%	0.72%

Notes: Notes: PHEV denotes Plug-in Hybrid Electric Vehicle, HEV hybrid vehicles, EV denotes electric vehicle., VT = vehicle tax, RT = road tax

Supplementary Note 8. Exponential rise in EV sales share

Our data (Marklines, IEA) on EV sales suggest an exponential rise in sales for 33 countries and worldwide up to 2022, but shows less than exponential growth for 2023. This is reflected in a recent IEA report.[37] While this could indicate a limitation to the diffusion of EVs, it is not representative of the long term trend visible in the data. The slowdown relates to the removal or reduction of subsidies in Germany and elsewhere. However, total numbers continue to grow, and may accelerate again as the policy landscape evolves.

Supplementary References

1. Farmer, J. D. & Lafond, F. How predictable is technological progress? *Res. Policy* (2016) doi:10.1016/j.respol.2015.11.001.
2. McNerney, J., Farmer, J. D., Redner, S. & Trancik, J. E. Role of design complexity in technology improvement. *Proc. Natl. Acad. Sci.* **108**, 9008–9013 (2011).
3. Yelle, L. E. Adding life cycles to learning curves. *Long Range Plann.* **16**, 82–87 (1983).
4. Utterback, J. M. & Abernathy, W. J. A dynamic model of process and product innovation. *Omega* **3**, 639–656 (1975).
5. Mercure, J.-F. FTT:Power : A global model of the power sector with induced technological change and natural resource depletion. *Energy Policy* **48**, 799–811 (2012).
6. Mercure, J.-F. An age structured demographic theory of technological change. *J. Evol. Econ.* **25**, (2015).
7. Mercure, J. F. Fashion, fads and the popularity of choices: Micro-foundations for diffusion consumer theory. *Struct. Change Econ. Dyn.* **46**, 194–207 (2018).
8. Mercure, J.-F., Lam, A., Billington, S. & Pollitt, H. Integrated assessment modelling as a positive science: private passenger road transport policies to meet a climate target well below 2 ◦C. *Clim. Change* (2018) doi:10.1007/s10584-018-2262-7.
9. Mercure, J. F. & Lam, A. The effectiveness of policy on consumer choices for private road passenger transport emissions reductions in six major economies. *Environ. Res. Lett.* **10**, 064008 (2015).
10. Lam, A. & Mercure, J.-F. Which policy mixes are best for decarbonising passenger cars? Simulating interactions among taxes, subsidies and regulations for the United Kingdom, the United States, Japan, China, and India. *Energy Res. Soc. Sci.* (2021) doi:10.1016/j.erss.2021.101951.
11. Anderson, S. P., De Palma, A. & Thisse, J. F. *Discrete Choice Theory of Product Differentiation*. (MIT press, 1992).
12. Arthur, W. B. & Lane, D. A. Information contagion. *Struct. Change Econ. Dyn.* **4**, 81–104 (1993).
13. McShane, B. B., Bradlow, E. T. & Berger, J. Visual influence and social groups. *J. Mark. Res.* **49**, 854–871 (2012).
14. Hofbauer, J. & Sigmund, K. *Evolutionary Games and Population Dynamics*. (Cambridge University Press, 1998).

15. Dargay, J., Gately, D. & Sommer, M. Vehicle Ownership and Income Growth, Worldwide: 1960-2030. *Energy J.* **28**, 143–170 (2007).

16. Dargay, J. & Gately, D. Income's effect on car and vehicle ownership, worldwide: 1960–2015. *Transp. Res. Part Policy Pract.* **33**, 101–138 (1999).

17. Cuaresma, J. C. Income projections for climate change research: A framework based on human capital dynamics. *Glob. Environ. Change* **42**, 226–236 (2017).

18. Gan, Y. *et al.* Future private car stock in China: current growth pattern and effects of car sales restriction. *Mitig. Adapt. Strateg. Glob. Change* **25**, 289–306 (2020).

19. Meyer, I., Kaniovski, S. & Scheffran, J. Scenarios for regional passenger car fleets and their CO2 emissions. *Energy Policy* **41**, 66–74 (2012).

20. Dimitropoulos, A., Oueslati, W. & Sintek, C. The rebound effect in road transport: A meta-analysis of empirical studies. *Energy Econ.* **75**, 163–179 (2018).

21. Hymel, K. M., Small, K. A. & Van Dender, K. Induced demand and rebound effects in road transport. *Transp. Res. Part B Methodol.* **44**, 1220–1241 (2010).

22. Small, K. A. & Dender, K. V. Fuel Efficiency and Motor Vehicle Travel: The Declining Rebound Effect. *Energy J.* **28**, 25–52 (2007).

23. Dahl, C. A. Measuring global gasoline and diesel price and income elasticities. *Energy Policy* **41**, 2–13 (2012).

24. Labandeira, X., Labeaga, J. M. & López-Otero, X. A meta-analysis on the price elasticity of energy demand. *Energy Policy* **102**, 549–568 (2017).

25. Karathodorou, N., Graham, D. J. & Noland, R. B. Estimating the effect of urban density on fuel demand. *Energy Econ.* **32**, 86–92 (2010).

26. Pettifor, H., Wilson, C., Axsen, J., Abrahamse, W. & Anable, J. Social influence in the global diffusion of alternative fuel vehicles--A meta-analysis. *J. Transp. Geogr.* **62**, 247–261 (2017).

27. Gillingham, K., Rapson, D. & Wagner, G. The Rebound Effect and Energy Efficiency Policy. *Rev. Environ. Econ. Policy* **10**, 68–88 (2016).

28. Scheffer, M. *et al.* Early-warning signals for critical transitions. *Nature* **461**, 53–59 (2009).

29. Lenton, T. M. *et al.* Tipping elements in the Earth's climate system. *Proc. Natl. Acad. Sci.* **105**, 1786–1793 (2008).

30. Armstrong McKay, D. I. *et al.* Exceeding 1.5°C global warming could trigger multiple climate tipping points. *Science* **377**, eabn7950 (2022).

31. Otto, I. M. *et al.* Social tipping dynamics for stabilizing Earth's climate by 2050. *Proc. Natl. Acad. Sci.* **117**, 2354–2365 (2020).

32. Lenton, T. M. Tipping positive change. *Philos. Trans. R. Soc. Lond. B. Biol. Sci.* (2020) doi:10.1098/rstb.2019.0123.

33. Sharpe, S. & Lenton, T. M. Upward-scaling tipping cascades to meet climate goals: plausible grounds for hope. *Clim. Policy* **21**, (2021).

34. Rogers, E. M. *Diffusion of Innovations*. (Simon and Schuster, 2010).

35. Arthur, W. B. Competing technologies, increasing returns, and lock-in by historical events. *Econ. J.* **99**, 116–131 (1989).

36. BNEF. *Hitting the EV Inflection Point*. (2021).

37. IEA. *EV Outlook 2024*. (2024).

38. LME. *Monthly Average Prices*. (2020).

39. Nykvist, B. & Nilsson, M. Rapidly falling costs of battery packs for electric vehicles. *Nat. Clim. Change* **5**, 329–332 (2015).

40. UBS. *UBS Evidence Lab Electric Car Teardown – Disruption Ahead?* https://neo.ubs.com/shared/d1wkuDlEbYPjF/ (2017).

41. Berckmans, G. *et al.* Cost Projection of State of the Art Lithium-Ion Batteries for Electric Vehicles Up to 2030. *Energies* **10**, 1314 (2017).

42. Kittner, N., Lill, F. & Kammen, D. M. Energy storage deployment and innovation for the clean energy transition. *Nat. Energy* **2**, 1–6 (2017).

43. Nykvist, B., Sprei, F. & Nilsson, M. Assessing the progress toward lower priced long range battery electric vehicles. *Energy Policy* **124**, 144–155 (2019).

44. Mauler, L., Duffner, F., G. Zeier, W. & Leker, J. Battery cost forecasting: a review of methods and results with an outlook to 2050. *Energy Environ. Sci.* **14**, 4712–4739 (2021).

45. Thiel, C., Perujo, A. & Mercier, A. Cost and CO2 aspects of future vehicle options in Europe under new energy policy scenarios. *Energy Policy* **38**, 7142–7151 (2010).

46. IEA. *Evolution of Li-Ion Battery Price, 1995-2019*. https://www.iea.org/data-and-statistics/charts/evolution-of-li-ion-battery-price-1995-2019 (2020).

47. ICCT. *Evaluating Electric Vehicle Costs and Benefits in China in the 2020–2035 Time Frame*. https://theicct.org/sites/default/files/publications/EV-costs-benefits-china-EN-apr2021.pdf (2021).

48. Van Velzen, A., Annema, J. A., van de Kaa, G. & van Wee, B. Proposing a more comprehensive future total cost of ownership estimation framework for electric vehicles. *Energy Policy* **129**, 1034–1046 (2019).

49. Allcott, H. & Wozny, N. Gasoline Prices, Fuel Economy, and the Energy Paradox. *Rev. Econ. Stat.* **96**, 779–795 (2014).

50. Busse, M. R., Knittel, C. R. & Zettelmeyer, F. Are consumers myopic? Evidence from new and used car purchases. *Am. Econ. Rev.* **103**, 220–256 (2013).

51. OPEC. *OPEC Basket Price.* (2022).

52. Eurostat. Electricity prices for non-household consumers - annual data (from 2007 onwards). (2020).

53. ODYSSEE-MURE. *Change in Distance Travelled by Car.* (2020).

54. National Bureau of Statistics of China. *China Statistical Yearbook*. (2021).

55. Office of Highway Policy Information. *US Highway Statistics*. (2020).
